# Outcomes of elective liver surgery worldwide: a global, prospective, multicenter, cross-sectional study

**DOI:** 10.1097/JS9.0000000000000711

**Published:** 2023-10-04

**Authors:** The LiverGroup.org Collaborative*

**Keywords:** failure to rescue, global surgery, human development index, liver surgery, morbidity, mortality, outcomes

## Abstract

**Background:**

The outcomes of liver surgery worldwide remain unknown. The true population-based outcomes are likely different to those vastly reported that reflect the activity of highly specialized academic centers. The aim of this study was to measure the true worldwide practice of liver surgery and associated outcomes by recruiting from centers across the globe. The geographic distribution of liver surgery activity and complexity was also evaluated to further understand variations in outcomes.

**Methods:**

LiverGroup.org was an international, prospective, multicenter, cross-sectional study following the Global Surgery Collaborative Snapshot Research approach with a 3-month prospective, consecutive patient enrollment within January–December 2019. Each patient was followed up for 90 days postoperatively. All patients undergoing liver surgery at their respective centers were eligible for study inclusion. Basic demographics, patient and operation characteristics were collected. Morbidity was recorded according to the Clavien–Dindo Classification of Surgical Complications. Country-based and hospital-based data were collected, including the Human Development Index (HDI). (NCT03768141).

**Results:**

A total of 2159 patients were included from six continents. Surgery was performed for cancer in 1785 (83%) patients. Of all patients, 912 (42%) experienced a postoperative complication of any severity, while the major complication rate was 16% (341/2159). The overall 90-day mortality rate after liver surgery was 3.8% (82/2,159). The overall failure to rescue rate was 11% (82/ 722) ranging from 5 to 35% among the higher and lower HDI groups, respectively.

**Conclusions:**

This is the first to our knowledge global surgery study specifically designed and conducted for specialized liver surgery. The authors identified failure to rescue as a significant potentially modifiable factor for mortality after liver surgery, mostly related to lower Human Development Index countries. Members of the LiverGroup.org network could now work together to develop quality improvement collaboratives.

## Introduction

Highlights

- International snapshot study following the global surgery collaborative approach.
- Over 2000 patients were included from all continents.
- The 90-day mortality rate after liver surgery was 4%.
- The failure to rescue rate ranged from 5 to 35% among higher and lower Human Development Index (HDI) groups.
- There is a great need to develop quality improvement collaboratives worldwide.

The most common indications for liver surgery include primary cancer, such as hepatocellular and cholangiocarcinoma, liver metastases, mostly colorectal, and benign liver lesions, including hydatid liver disease. Asia and the Western Pacific have the highest liver malignancy mortality rates nearing 20%, especially in upper-middle-income areas, while the lowest rates are observed in South America, Europe, South-East Asia, as well as in low-middle-income regions^1^. Liver cancer deaths were over 800 000 in 2020, with the highest rates observed in Eastern Asia and Northern Africa^2^. Furthermore, it has was shown that socioeconomic factors have a significant impact on liver cancer outcomes^3^. The Human Development Index (HDI) is a metric of life expectancy, education, and standard of living, developed to assess social and economic differences among countries^4^. This metric is therefore useful to compare outcomes after liver surgery in different regions across the world.

Apart from being the only potentially curative approach for many liver malignancies^5^, hepatobiliary surgery is often a vital intervention in managing benign liver diseases, such as large symptomatic liver cysts, hepatic adenomas, and hydatid liver disease, among others^6^. Liver surgery ranges vastly in degrees of complexity and relies on strong cohesive multidisciplinary care. However, access to safe surgery of the liver worldwide is yet to be addressed. It is in this context that identifying the gaps in global surgery of the liver can help pinpoint modifiable domains in healthcare provision and perioperative care to improve the accessibility and safety of surgery worldwide. A light was shed on the gross inequality in surgery and anesthesia care in the world by the former President of the World Bank, Jim Yong Kim, in his statement to promote the inclusion of surgical care in the global health agenda that ʻsurgical care is an indivisible, indispensable part of healthcareʼ^7^. As the need to upscale healthcare systems, infrastructure, and education in underserved countries is increasingly evident, global surgery, a field of research and advocacy to improve surgical care outcomes not restricted to specific diseases, populations, or geographical regions, is of mounting significance today^8,9^. Thus, global surgery research should not only focus on ʻessentialʼ, cost-effective general surgical procedures^10^ but also on highly specialized surgery, such as hepatobiliary.

Ultimately, the true demographics worldwide in terms of activity and outcomes of hepatobiliary surgery remain unknown and surgery of the liver as we know it reflects the activity of highly specialized academic centers. In addition, the true population-based outcomes are likely different to those vastly reported from high-volume academic centers^11–13^. A comprehensive understanding of the global necessity for surgical interventions is paramount to address the significant health challenges faced by populations worldwide. However, it is equally crucial to acknowledge and analyze the barriers to accessing surgical care, including workforce shortages and quality concerns, to effectively enhance global surgical standards. The importance of this endeavor lies in its potential to mitigate disparities and optimize hepatobiliary surgical outcomes across diverse healthcare settings^14^.

In this International Liver Surgery Outcomes Study, the primary objective was to measure the true worldwide practice of liver surgery and associated outcomes by recruiting from centers across the globe, committing to consecutive case registration and rigorous data validation. The geographic distribution of liver surgery activity and complexity was also evaluated to further understand variations in outcomes.

## Methods

### Study design

LiverGroup.org was an international, prospective, multicenter, cross-sectional study following the Global Surgery Collaborative Snapshot Research approach which was introduced in the UK in 2013^15^. Such research methodology provides a worldwide population-based overview of the current clinical practice and allows for hypothesis generating comparative analyses. The study protocol was registered in advanced at ClinicalTrials.gov (NCT03768141) and audit approval was obtained by the Royal Free Hospital London, UK. This work is reported in line with the strengthening the reporting of cohort, cross-sectional and case–control studies in surgery (STROCSS) criteria^16^ (Supplemental Digital Content 3, http://links.lww.com/JS9/B128).

### Study interval

The project and study design were initiated in May 2017, center recruitment in September 2018, with a 3-month of prospective, consecutive patient enrollment within January to December 2019. Each patient was followed up for up to 90 days postoperatively. None of the participating centers were affected by the first peak of COVID-19 during the study period^17^.

### Center inclusion and recruitment

Any surgeon performing liver surgery was eligible to participate and there were no exclusion criteria for center participation, to reflect the nature of this global surgery study. Centers were recruited through various methods, including: collaborative networks and partnerships with healthcare institutions, through national societies of surgical trainees, through different associations and societies by promoting our study to their members, through research collaborations with centers that had prior projects with the study investigators, through preselected country and regional leaders dedicated to recruit centers within their region, through dissemination of study information at conferences, through multiple emails and newsletters, social media, instant messaging groups, by utilizing personal contacts, and referrals within the global surgery community.

### Participants and procedures

All patients undergoing liver surgery at their respective centers were eligible for study inclusion. The inclusion criteria were patients of 18 years of age or older, any indication for surgery, including surgery for both benign and malignant disease, and all minimally invasive approaches. The exclusion criteria were patients undergoing liver transplantation, liver biopsies, or image-guided liver ablation alone. For this manuscript, only single-stage liver resections were included, two-stage liver resection^18^ for the purposes of liver parenchyma augmentation were excluded. It was predetermined at the beginning of the study design that data on two-stage liver resections would warrant separate examination and documentation. The purpose of the present study was to report the true morbidity and mortality associated with liver surgery. Only reason I would delete this is because it sounds like a bias introduced rather than a measure to minimise it (which I believe was the intention).

### Data

Basic demographics, patient and operation characteristics were collected. The complexity of liver surgery was defined according to certain criteria and each criterion was given a single point (maximum 13) to create the Liver Surgery Complexity Score. Morbidity was recorded according to the Clavien–Dindo Classification of Surgical Complications^19^, the FABIB Liver Surgery-Specific Classification^20^ and the novel Comprehensive Complication Index (CCI)^21^ up to 90 days postoperatively. Major complications were defined as Clavien–Dindo grade ≥3a (any complication requiring an intervention or organ failure). High-volume centers were defined as those having submitted at least 30 cases over the 3-month recruitment period (extrapolating it to 120 cases annually). The failure-to-rescue rate was calculated by dividing the number of patients that died after surgery over the total number of patients with complications^22^. All data were collected using the LiverGroup.org specially designed electronic Case Report Form (eCRF). Furthermore, country-based and hospital-based data were collected, including the Human Development Index (HDI)^4^, Gross National Income (GNI)^23^, Education Index^23^, Life Expectancy Index (LEI)^23^, and the Total Health Expenditure^24^. These parameters were used to compare the outcomes of liver surgery worldwide. Cost analysis was conducted using the validated AssesSurgery GmbH calculator^25^ only for European countries, including Switzerland and the United States of America. All values in Euro or Swiss Francs were converted to US Dollars for comparisons and uniform reporting purposes.

### Power considerations

This study aimed for the maximum number of patients able to recruit. Assuming a 90-day mortality rate of 5 and 50% reduction to 2.5%, an alpha error of 0.05, power of 80%, the sample size calculation revealed the need for 1828 patients to be recruited. When adjusting for 10% drop-out or missing data rate, the final sample size calculation was set at 2011 cases in total.

### Statistical methods

Continuous variables were compared with the Student *t*-test, the Mann–Whitney *U* test and the Kruskal–Wallis *H* test or one-way ANOVA where appropriate. Differences among proportions derived from categorical data were compared using the Fisher and the Pearson *χ* ^2^-tests, where appropriate. The Complexity of Liver Surgery Score (maximum score 13) was internally validated using the intraclass collection coefficient and the Cronbach’s alpha test. ROC curve analysis was used to assess its predictive value and the Yuden’s index to identify the optimal score cut-off point. A multivariable, binary regression analysis was performed to identify independent factors of 90-day mortality. The nature of missingness and proportions of missing data per variable were assessed. Variables containing data *missing completely at random* and missing in fewer than 10% of observations were handled as complete case analysis^26^. Cases with missing outcome only (morbidity and mortality) data were excluded and there were no attempts to perform multiple imputation calculations to replace them. All *P*-values were 2-sided and considered statistically significant if *P*<0.05. Statistical analysis was performed using R version 3.3.2 (R Core Team, GNU GPL v2 License), R Studio version 1.0.44 (RStudio, Inc. GNU Affero General Public License v3, 2016) with the graphical user interface (GUI) rBiostatistics.com (rBiostatistics.com, 2017).

## Results

### Participants

**Figure 1:**
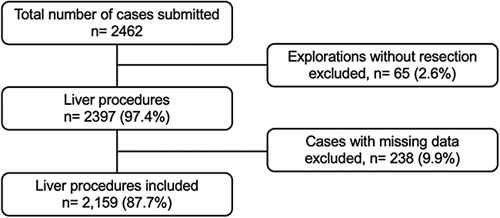
Flow diagram of included cases in the study. Cases with outcome data (morbidity and mortality) were excluded from the analysis.

**Figure 2:**
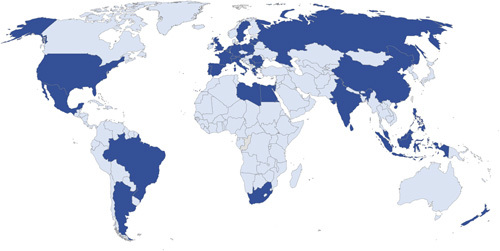
Map of participating centers worldwide. Dark blue indicates participation.

A total of 2159 patients were included (Fig. 1) from six continents, 36 countries (Fig. 2), and 136 institutions. Demographics, disease, and operation characteristics are reported in Tables 1 and 2.

**Table 1:** Patient and disease characteristics.

Parameters	Values
Patient Characteristics	
Age, median (IQR)	64 (54–71)
Female sex, *n* (%)	1016 (43)
Race, *n* (%)	
African	38 (2)
Asian	159 (7)
Caucasian	1887 (87)
Other	75 (4)
BMI kg/m^2^, median (IQR)	26 (23–29)
Comorbidities, *n* (%)	
Coronary artery disease	237 (10)
Heart failure	85 (4)
Diabetes mellitus	383 (16)
Metastatic cancer	664 (27)
Hepatitis B or C	194 (9)
Liver cirrhosis	164 (7)
Stroke	45 (2)
COPD/Asthma	170 (7)
Other	747 (34)
Disease Characteristics, *n* (%)	
Malignancy	2050 (84)
Cholangiocarcinoma - hilar	113 (5)
Cholangiocarcinoma - intrahepatic	161 (7)
Colorectal liver metastases	1070 (43)
Hemangioma	60 (2)
Hepatic adenoma	48 (2)
Hepatocellular carcinoma	410 (17)
Nodular regenerative hyperplasia	1 (0)
Noncolorectal liver metastases	174 (7)
Sarcoma	9 (0)
Other	409 (16)
Sequence of surgery, *n* (%)	
Metachronous	398 (48)
Synchronous - combined	90 (11)
Synchronous - liver first	85 (10)
Synchronous - primary first	244 (30)
Previous therapy, *n* (%)	
Preoperative chemotherapy	753 (35)
Biological agents used	301 (14)
Previous abdominal surgery	1215 (56)
Previous liver resection	341 (14)
Liver specific characteristics	
Diameter of the largest lesion, median (IQR)	27 (16–43)
Liver parenchyma, *n* (%)	
Normal	802 (37)
Fibrosis	187 (9)
Cirrhosis	183 (9)
Steatosis	267 (12)
Chemotherapy induced injury	252 (12)
PVE prior to resection, *n* (%)	92 (4)
sFLR prior to resection, median %, (IQR)	42 (34–60)
sFLR prior to resection, mean %, (SD)	49 (21)

**Table 2:** Operation characteristics.

Parameters	Values
Mode of resection, *n* (%)	
Open	1777 (75)
Laparoscopic	513 (22)
Robotic	42 (2)
Hybrid / converted to open	42 (2)
Operation duration in min., median (IQR)	220 (150–300)
Operation performed, *n* (%)	
Ablation only	23 (1)
Bisegmentectomy	116 (5)
Left hepatectomy	228 (10)
Left lateral sectionectomy	190 (8)
Left trisectionectomy	30 (1)
Nonanatomical resection	873 (37)
Right hepatectomy	372 (16)
Right posterior sectionectomy	126 (5)
Right trisectionectomy	81 (3)
Segmentectomy	309 (13)
Trisegmentectomy	25 (1)
Complexity of liver surgery, *n* (%)	
Hilar lymphadenectomy	285 (13)
More than one liver resection	410 (19)
Resection and ablation	94 (4)
Portal vein resection and reconstruction	41 (2)
Hepatic vein resection and reconstruction	24 (1)
Hepatic artery resection and reconstruction	12 (1)
Vena vava resection and reconstruction	19 (1)
Bile duct resection and extrahepatic reconstruction	81 (4)
Bile duct resection and intrahepatic reconstruction	50 (2)
Enteric resection and reconstruction	132 (6)
Extrahepatic nongastrointestinal resection	62 (3)
Ante situ perfusion and resection	2 (0)
Ex situ perfusion and resection	*0* (*0)*
Complexity of liver surgery score, mean (SD)	0.6 (0.8)
Vascular exclusion, *n* (%)	
On-demand pringle	433 (20)
Intermittent pringle	502 (24)
Pringle and inferior vena cava clamping	13 (1)
Total vascular exclusion	19 (1)
Total clamp time in min., median, (IQR)	25 (12–40)
Transection technique, *n* (%)	
Crush clamp	294 (14)
*CUSA / Dissectron*	*1348* (*62)*
Bipolar	822 (38)
Stapler	300 (14)
Other	592 (27)
Transection time in min., median (IQR)	60 (35–100)
Other information	
Blood transfusions, mean (SD)	0.4 (1.3)
Extubated in the operation room, n (%)	1434 (80)

Surgery was performed for cancer in 1785 (82.7%) patients, of whom 296 (13.7%) had received a liver resection previously. In 914 (42.3%) patients the indication for resection was colorectal liver metastases (CRLM), while in 386 (17.9%) it was hepatocellular carcinoma (HCC) (Table 1). Minimally invasive surgery was performed in 512 patients (23.7%) patients (Table 2). A total of 781 (36.3%) underwent liver wedge (nonanatomical) resections. Additional to surgery, intraoperative ablations of liver lesions were performed in 136 (6.3%) of patients. The overall mean Complexity of Liver Surgery Score was 0.6 (SD 0.8), ranging from 0 to 6. This score could discriminate morbidity and mortality in a relatively linear manner; higher the score, higher the morbidity (ranging from 11% to 75%) and mortality (3% to 29%, respectively, *P*<0.001) rates were at 90 days postoperatively (Supplementary Figure 14, Supplemental Digital Content 1, http://links.lww.com/JS9/B126). Similarly, on multivariable analysis, the complexity of the liver sugary score, with a cut-off of 2 points (Supplementary Figure 15 Supplemental Digital Content 1, http://links.lww.com/JS9/B126), was identified as an independent predictor of mortality (OR 3.83, 95% CI: 2.27–6.37, *P*<0.001) (Supplementary Figure 16 Supplemental Digital Content 1, http://links.lww.com/JS9/B126). Figure 4.

**Figure 3:**
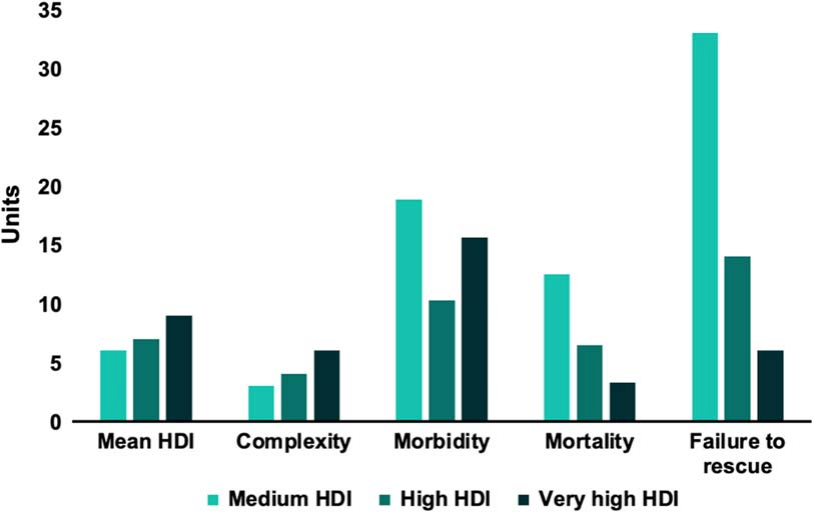
Complexity of liver surgery, morbidity, mortality, and failure to rescue among the 3 HDI groups (medium, high, and very high). Mean HDI and complexity scores were multiplied by 10 for visualization purposes. Morbidity, mortality, and failure to rescue rates represent percentages.

**Figure 4:**
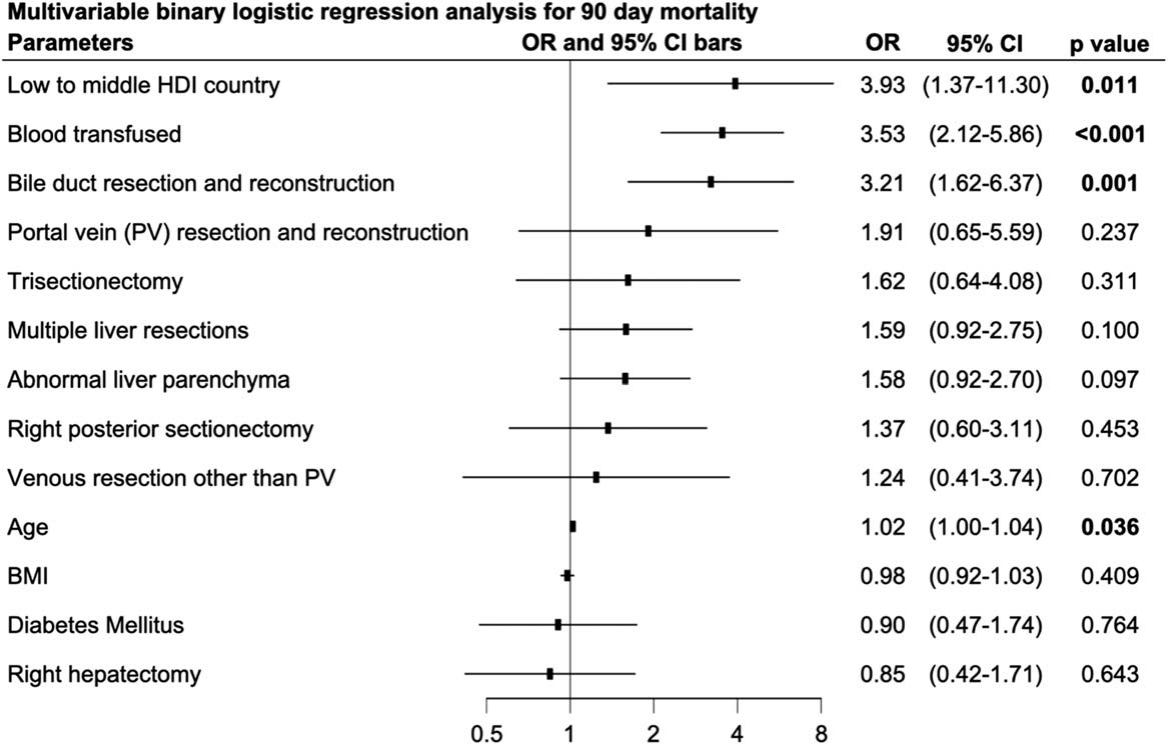
Multivariable analysis for independent factors of 90-day mortality, including the low- to middle HDI group.

In 211 (9.8%) patients, liver resection was performed together with a form of biliary and/or venous reconstruction. The median intraoperative and first 24 h postoperatively packed red blood cell (RBC) transfusion was 0 (10th–90th percentile: 0–1). Finally, a total of 1434 (80.1%) patients were successfully extubated in the operation room (Table 2).

### Outcomes

Outcomes were measured from the completion of surgery up until 90 days postoperatively. Of all patients, 912 (42.2%) experienced a postoperative complication of any severity, while the major complication rate was 15.8% (341/2,159) (Tables 3 and 4). Organ failure occurred in 88 (4.1%), ascites (Grade 2 or higher) in 130 (6.0%), bile leak in 182 (8.4%), infection in 344 (15.9%), and bleeding in 140 (6.5%) of the patients (Tables 3 and 4). The median ICU and hospital stay was 1 (IQR 0–2) and 8 (5–11) days, respectively. The median hospital readmission rate was 11.1% (240/2159) up until 90 days postoperatively. The overall 90-day mortality rate after liver surgery was 3.8% (82/2159). The mortality rate among high-volume participating centers ranged from 0 to 9% (Supplementary figure 1 Supplemental Digital Content 1, http://links.lww.com/JS9/B126). Morbidity and mortality rates according to the different indications and types of operations are reported in Table 3. Of note, the 90-day postoperative mortality varied significantly among indications for surgery, with colorectal cancer metastases being 2% while for hilar cholangiocarcinoma as high as 19%. Furthermore, although there were significant differences in baseline characteristics and overall complication rates, major morbidity (14 vs. 16%, OR 1.14 (95% CI: 0.82–1.59, *P*=0.483) and mortality (3 vs. 4%, OR 1.52 (95% CI: 0.77–3.33, *P*=0.295) did not differ between benign and malignant indications for liver surgery (Supplemental Table 1, Supplemental Digital Content 2, http://links.lww.com/JS9/B127).

**Table 3:** Morbidity and mortality rates according to the different indications and types of operations.

Parameters	Complexity score, mean (SD)	Grade >2, n, (%)	Mortality, n, (%)
Indication			
Hilar cholangiocarcinoma	2.0 (1.0)	38/88 (43)	16/85 (19)
Intrahepatic cholangiocarcinoma	0.8 (1.0)	39/151 (26)	7/147 (5)
Colorectal liver metastases	0.6 (0.7)	116/914 (13)	22/898 (2)
Focal nodular hyperplasia	0.1 (0.3)	6/32 (19)	1/32 (3)
Hemangioma	0.1 (0.3)	3/58 (5)	3/55 (6)
Hepatic adenoma	0.1 (0.3)	1/44 (2)	1/45 (2)
Hepatocellular carcinoma	0.3 (0.5)	58/386 (15)	16/382 (4)
Noncolorectal liver metastases	0.7 (0.9)	20/141 (14)	1/147 (1)
Gallbladder cancer	0.9 (0.7)	12/64 (19)	7/62 (11)
Surgical approach			
Open	0.7 (0.9)	303/1647 (18)	70/1625 (4)
Hybrid/converted to open	0.5 (0.9)	10/42 (24)	2/41 (5)
Laparoscopic	0.3 (0.5)	26/428 (6)	10/413 (2)
Robotic	0.1 (0.3)	2/42 (5)	0/42 (0)
Type of operation			
Ablation only	0.0 (0.0)	0/11 (0)	0/11 (0)
Bisegmentectomy	0.6 (0.7)	15/116 (13)	7/113 (6)
Left hepatectomy	0.8 (1.0)	43/215 (20)	11/208 (5)
Left lateral sectionectomy	0.4 (0.7)	15/190 (8)	5/187 (3)
Left trisectionectomy	1.0 (1.0)	11/28 (39)	3/28 (11)
Nonanatomical resection	0.5 (0.7)	91/781 (12)	16/765 (2)
Right hepatectomy	0.5 (0.7)	69/349 (20)	15/348 (4)
Right posterior sectionectomy	0.7 (1.0)	32/126 (25)	9/123 (7)
Right trisectionectomy	1.0 (1.0)	32/71 (45)	5/71 (7)
Segmentectomy	0.3 (0.6)	26/241 (11)	10/238 (4)
Trisegmentectomy	1.0 (1.0)	7/25 (28)	1/25 (4)
Complexity of liver surgery			
No complexity features	–	173/1411 (12)	43/1385 (3)
Hilar lymphadenectomy	–	92/285 (32)	24/277 (9)
More than one liver resection	–	68/410 (17)	22/406 (5)
Resection and ablation	–	12/94 (13)	4/93 (4)
Portal vein resection and reconstruction	–	20/41 (49)	8/40 (20)
Hepatic vein resection and reconstruction	–	12/24 (50)	2/24 (8)
Hepatic artery resection and reconstruction	–	6/12 (50)	2/12 (17)
Vena cava resection and reconstruction	–	9/19 (47)	3/19 (16)
Bile duct resection and extrahepatic reconstruction	–	41/81 (51)	14/78 (18)
Bile duct resection and intrahepatic reconstruction	–	23/50 (46)	4/49 (8)
Associated enteric resection and reconstruction	–	39/132 (30)	8/130 (6)
Extrahepatic non gastrointestinal resection	–	18/62 (29)	1/62 (2)

**Table 4:** Postoperative outcomes.

Parameters	Values
Clavien–Dindo highest grade, *n* (%)	
No complications	1394 (58)
Grade 1 – no treatment	214 (9)
Grade 2 – pharmacological treatment	420 (18)
Grade 3a – intervention under LA	185 (8)
Grade 3b – intervention under LA	86 (4)
Grade 4a – single organ failure	42 (2)
Grade 4b – multi-organ failure	10 (1)
Grade 5– death	46 (2)
Clavien–Dindo grades grouped, *n* (%)	
Any complication	912 (42)
Grade >1	722 (33)
Grade >2	341 (16)
Grade >3a	170 (8)
FABIB Classification, *n* (%)	
Failure	
None	2308 (96)
A	42 (2)
B	25 (1)
C	22 (1)
Overall	89 (4)
Ascites	
None	2265 (94)
A	100 (4)
B	27 (1)
C	5 (0)
Overall	132 (6)
Bile leak	
None	2209 (92)
A	71 (3)
B	87 (4)
C	30 (1)
Overall	187 (8)
Infection	
None	2045 (85)
A	226 (9)
B	93 (4)
C	33 (1)
Overall	370 (15)
Bleeding	
None	2253 (94)
A	84 (4)
B	30 (1)
C	30 (1)
Overall	144 (6)
Other postoperative outcomes	
Intensive care unit stay, median (IQR)	1 (0–2)
Hospital stay in days, median (IQR)	8 (5–11)
Hospital readmission rate, *n* (%)	240 (10)
Mortality rate, *n* (%)	87 (3.7)
Centre adjusted mortality rate, median (IQR)	2.1 (0–3.8)

The overall failure to rescue rate was 11.4% (82/ 722) (dead/complications). The overall mean estimated cost of liver surgery in Europe and the USA was 14 034 (SD 7279) US Dollars. Supplementary figure 2 Supplemental Digital Content 1, http://links.lww.com/JS9/B126 illustrates the increasing estimated cost per different grades of postoperative complications. Briefly, the cost of complications approximately double with organ failure requiring ITU admission and triple when complications lead to death.

### Hospital characteristics

The mortality rate in relatively small (<850 beds) *versus* large (≥850 beds) hospitals was not significantly different in this cohort (4.4 vs. 3.4%, OR 1.31, 95% CI: 0.82–2.10, *P*=0.261). Teaching or university affiliated hospitals; however, were associated with lower mortality rates when compared to nonteaching hospitals (3.5 vs. 7.4%, OR 0.45, 95% CI: 0.25–0.87, *P*=0.011). There was a trend to higher mortality rate associated with private when compared to public hospitals 3.7 vs. 7.9%, OR 2.23, 95% CI: 0.84–5.03, *P*=0.081).

### Patient, operative characteristics, and outcomes across the human development index groups

The Human Development Index (HDI) (Supplementary figure 3 Supplemental Digital Content 1, http://links.lww.com/JS9/B126) is a summary measure of achievement in key dimensions of human development: Life Expectancy (LEI), Education (EI) and the Gross National Income (GNI) Indices^23^. All participating centers fell into three groups, the low- to medium- (0.550–0.699), high- (0.700–0.799) and very high- (≥0.800) HDI groups. Although sex and comorbidities did not differ significantly among the three HDI groups, patients from low- to middle HDI countries were younger, were more likely to suffer from a benign disease, had similar postoperative morbidity rates but despite that, a significantly higher mortality rate when compared to high- and very high-HDI groups (13, 7, and 4%), respectively (*P*=0.004). Figure 3 illustrates the different complexity of liver surgery scores, morbidity, failure to rescue, and mortality rates among the three HDI groups. Of note, complexity increased while failure to rescue and mortality rates decreased per higher HDI group. Similar trends arose when analyzing LEI, EI, and GNI, and other indices separately (Table 5, Supplementary Figures 4–13 Supplemental Digital Content 1, http://links.lww.com/JS9/B126).

**Table 5:** Characteristics of patients within the human development index (HDI) groups.

	Low to medium HDI	High-HDI	Very- high#HDI	
Parameters	*n=*48	*n=*126	*n=*1985	*P*
Age, median (IQR)	48 (35–63)	54 (36–64)	65 (55–72)	**<0.001**
Female sex, *n* (%)	23 (48)	50 (40)	863 (44)	0.574
BMI kg/m^2^, median (IQR)	23 (22–27)	25 (22–27)	26 (23–29)	0.113
Coronary artery disease, *n* (%)	3 (6)	15 (12)	211 (11)	0.553
Diabetes mellitus, *n* (%)	10 (21)	21 (17)	337 (17)	0.776
Metastatic cancer, *n* (%)	21 (44)	86 (68)	1678 (68)	**<0.001**
Hepatitis B or C, *n* (%)	4 (8)	28 (14)	162 (8)	**<0.001**
Liver cirrhosis, *n* (%)	5 (10)	9 (7)	145 (7)	0.714
COPD/Asthma, *n* (%)	2 (4)	6 (5)	144 (7)	0.418
Malignancy, *n* (%)	21 (44)	86 (68)	1678 (85)	**<0.001**
Complexity of liver surgery, mean (SD)	0.3 (0.7)	0.4 (0.7)	0.6 0.8	**<0.001**
Minimally invasive approach, *n* (%)	18 (38)	32 (25)	464 (23)	**<0.001**
Operation duration in min., median (IQR)	360 (251–492)	300 (205–360)	214 (150–300)	**<0.001**
Blood transfusion units, mean, (SD)	0.9 (2)	0.5 (1)	0.3 (1)	**0.007**
Extubated in the operation room, *n* (%)	39 (89)	78 (71)	1317 (81)	**0.018**
Complication of any severity, 90 days	28 (58)	51 (41)	833 (42)	0.070
Grade ≥3a complication, 90 days	48 (19)	126 (10)	1985 (16)	0.195
Grade ≥3b complication, 90 days	4 (8)	7 (6)	159 (8)	0.607
CCI until 90 days postoperatively, median (IQR)	9 (0–21)	0 (0–11)	0 (0–21)	0.074
Intensive care unit stay, median (IQR)	3 (2–4)	1 (0–2)	1 (0–2)	0.133
Hospital stay in days, median (IQR)	8 (5–9)	10 (6–15)	8 (5–11)	0.214
Mortality rate, 90 days, *n* (%)	6 (13)	8 (7)	68 (4)	**0.004**

Statistically significant values are in bold.

## Discussion

The ʻtrueʼ picture of liver surgery that this snapshot study allows us to depict, reflects the current demographic distribution of activity and outcomes worldwide. Namely, regions of higher Human Development Index (HDI, a composite metric of life expectancy, education, and per capita income) perform an increasing number of liver resections, while Sub-Saharan Africa is grossly underrepresented. Liver surgery today bears important morbidity overall, with as many as 4 in 10 patients experiencing at least one postoperative complication of any severity with an overall 90-day mortality rate of 4%.

Across the represented HDI groups, there was an inverse relationship between increasing HDI and 90-day mortality rates with a four-fold higher mortality in the medium HDI group than in the very high-HDI group. Major morbidity, however, while also highest in the medium HDI group, was lowest in the high-HDI countries rather than in the very high-HDI countries. Interestingly, despite the very high-HDI countries owning the lowest mortality, the major morbidity was substantial. When evaluating the surgical complexity scores, it becomes apparent that the high morbidity is associated with higher complexity. The resulting relationship of these outcome metrics, particularly in the medium and very high-HDI group, rises further themes for discussion and research.

Failure-to-rescue, defined as death after a treatable complication, was described as an effective measure of preventable postoperative mortality^22,27^. This global snapshot study reveals a failure-to-rescue rate of over 10% in liver surgery worldwide. The stark contrast between the mortality rates between medium and very high-HDI groups, considering the relatively similar morbidity rates, is highly suggestive of higher preventable mortality in lower HDI groups. Similarly, in the African Surgical Outcomes Study (ASOS), a high incidence of preventable deaths in low-risk patients following surgery was identified and attributed to inadequate identification and treatment of life-threatening complications during the perioperative period^28^. Furthermore, several Global Surgery studies have emphasized the importance of failure to rescue as one of the few factors that are modifiable^29,30^. This has led to its use as a new quality indicator of surgical services^31^. Although the key modifiable domains that impact mortality following major complications in liver surgery remain hypothesis driven, the timely recognition and management of complications together with optimal infrastructure can hardly be disputed. Nationwide studies, albeit all from very high-HDI countries, have attributed interhospital differences in failure to rescue rates after liver surgery, at least in part, to hospital volume^32,33^. However, at an international level, there appears to be no significant association between the two, with hospital size and volume not being associated with postoperative outcomes.

The burden of postoperative mortality after surgery, of any kind, is estimated to account for nearly 10% of all deaths each year worldwide, which makes it the third leading cause of deaths, preceded only by ischemic heart disease and stroke^34^. Improving surgical care must embrace initiatives to reduce postoperative deaths as much as to address the disparities in surgical activity in underserved areas. While the present study reveals gaps in the field of liver surgery, failure to rescue encompasses the wider surgical ecosystem, including workforce and infrastructure factors. Identifying what drove the improved standards in the very high-HDI countries that have also allowed for resections of higher complexity will help address the high failure to rescue rates observed in the medium HDI group. In establishing an international collaborative group, LiverGroup.org members could now work together to develop quality improvement collaboratives.

This study also revealed other important findings. Liver surgery is currently performed bloodless, with only about 1 out of 10 patients requiring a blood transfusion. This is in contrast with historic reports that associated liver surgery with significant blood loses^35^. This may be attributed to improved surgical techniques and precision instruments, preoperative planning and advanced imaging technologies, inflow occlusion^36^, hemostatic agents and devices for effective bleeding control, optimized transfusion strategies and blood management protocols, as well as enhanced perioperative care practices. However, mortality rates reaching nearly 20% in patients that suffer from hilar cholangiocarcinoma, requiring complex liver surgery, biliary and/or venous reconstruction are unacceptable and twice as high when compared to the current literature^37^. Further research in this field is required to make surgery for cholangiocarcinoma safer, among others including risk stratification and patient selection scores, perioperative care optimization protocols, quality improvement initiatives, multicenter collaborations, and data registries.

A third of the patients underwent parenchymal preserving operations (i.e. nonanatomical resection, also known as wedges). This is in contrast to previous reports with anatomical, nonparenchymal preserving operations performed due to the perceived oncological benefit and simplicity of liver surgery^38^. Furthermore, minimally invasive surgery was attempted and/or performed in a quarter of the patients in this cohort, however the levels of surgical complexity in this group were lowest, which may explain superior outcomes. Interestingly, worse outcomes were associated with operations that were converted from minimally invasive to open surgery. This may reflect higher complexity as well as intraoperative complications affecting patient outcomes^39^.

Apart from the patient and family burden, postoperative complications also affect cost and hospital resources^40^. In-hospital cost appear to double for reinterventions and quadruple with organ failure. The highest costs were related to patients in whom failure-to-rescue occurred. Prevention and early identification of postoperative complications may help increase patient turnover as well as save cost, allowing to offer surgery to more patients and reduce the waiting time for surgery.

The strengths of this study lie in the magnitude of the LiverGroup.org network, its geographic distribution, the prospective nature of this study, the duration of follow-up, and the liver surgery-specific details obtained. Nevertheless, a study of this scale has some inevitable limitations. *Firstly*, selective reporting is an issue with any type of global surgery studies^41^ and there were no data monitors assigned to each center. However, with anonymous reporting and the high mortality rates associated with specific disease and operation characteristics, the writing committee members did not consider this as a significant issue. *Secondly*, the short time frame of three months for data capture by local investigators may risk selection bias, such as seasonal variation in local presentations. However, longer enrollment strategies, with a higher time burden, may have affected study participation. *Thirdly*, this study used several classifications and terminology that may not be familiar to local investigators, and this may have affected the correctness of data capture. However, the electronic Case Report Form (CRF) as well as the LiverGroup.org platform contained explanations for each classification and term used as well as online converters for laboratory values and other important calculators. *Fourthly*, the surgeon experience and learning curve was not assessed in this study, thus no associations could be made with regards to outcomes, especially among the different HDI country groups. *Lastly*, underrepresentation of certain regions in global surgery studies is a common phenomenon and this can be attributed to limited research infrastructure and funding opportunities in certain regions, lack of awareness and access to global surgery studies, language and cultural barriers that impact participation, variation in regulatory and ethical considerations across countries, as well as differences in surgical capacity and expertise among regions. Addressing these barriers requires initiatives to promote inclusivity and equity, such as capacity-building programs, targeted funding support, collaborations with regional partners, translation, and cultural adaptation of study materials, and active engagement with underrepresented regions to overcome specific challenges they may face^42^.

In conclusion, to our knowledge, this is the first global surgery study specifically designed and conducted for specialized, liver surgery. We identified failure to rescue as a significant potentially modifiable factor for mortality after liver surgery, mostly related to lower Human Development Index (HDI) countries. Members of the LiverGroup.org network could now work together to develop quality improvement collaboratives, with the next obvious step being studying failure to rescue in lower HDI countries. We propose a strategy encompassing joint research on failure-to-rescue factors in lower HDI countries, skill-based training programs, technology transfer, infrastructure development, policy advocacy, and local capacity-building

## Ethical approval

This project obtained audit approval from the Royal Free Hospital Audit and Compliance, Quality Governance department with the registration ID: RFH TASS40_2016/17.

## Consent

This project, registered as an audit, did not require informed consent in the UK as it was truly observational with fully anonymised data.

## Sources of funding

This study was supported by the University College London, UK Liver Fund Charity and BOWA AG, Germany. The sponsors how NO involvement in the collection, analysis and interpretation of data, in the writing of the manuscript, and in the decision to submit the manuscript for publication.

## Author contribution

DA.R., C.H.S., and M.M.: study conception and study design, data acquisition, data analysis and interpretation, article drafting and approval. All members of the Scientific Committee (see Appendix) revised and approved the manuscript for publication.

## Conflicts of interest disclosure

The authors declare that they have no financial conflict of interest with regard to the content of this report.

## Research registration unique identifying number (UIN)

ClinicalTrials.gov NIH Protocol (NCT03768141).

ISRCTN Registry Protocol (ISRCTN14071325).

## Guarantor

Dimitri Raptis and Massimo Malagò.

## Data availability statement

The Scientific and Management Committees will decide about requests from LiverGroup.org Members regarding data sharing and will consider all such requests based on quality and the validity of the proposed project.

## Provenance and peer review

Not commissioned, externally peer-reviewed.

## Appendix – Group Authorship

**LiverGroup.org Committees**

**Chief Investigators:**

Massimo Malagò, King Faisal Specialist Hospital & Research Centre, Riyadh, Saudi Arabia and Alejandro Serrablo, Hospital Universitario Miguel Servet, Spain. **Co-Chief Investigator**: Dimitri Aristotle Raptis, King Faisal Specialist Hospital & Research Centre, Riyadh, Saudi Arabia

**Scientific Committee:**

Christos Dervenis, Metropolitan Hospital Athens, Greece & University of Cyprus; Karl Jürgen Oldhafer, Asklepios Medical School, Germany; Marcel Autran Machado, Hospital Sirio Libanes, Brazil; Norihiro Kokudo, University of Tokyo Hospital, Japan, Pål-Dag Line, Oslo University Hospital, Norway; Roberto Hernandez-Alejandro, University of Rochester Medical Center, USA; Stefan Breitenstein, Cantonal Hospital Winterthur, Switzerland; Thomas van Gulik, Academic Medical Center, University of Amsterdam, Netherlands​; Yaman Tokat, International Liver Center, Turkey​ and Ulf Peter Neumann, University Hospital Aachen, Germany.

**Management Committee:**

Aditya Borakati, Royal Free Hospital London, UK; Andrea Monaco, Royal Free Hospital London, UK; Andreas A. Schnitzbauer, University Hospital Frankfurt, Germany; Arthur Elfrink​, University Medical Center Groningen​, Netherlands; Carlijn Buis, University Medical Center Groningen​, Netherlands; Camila Hidalgo Salinas, Royal Free Hospital London, UK; Charles Imber, Royal Free Hospital London, UK; Daniele Ferraro, Royal Free Hospital London, UK; Deniz Balci, Ankara University, Turkey; Dimitri Aristotle Raptis, Royal Free Hospital London, UK; Erik Schadde, Rush University Medical Center, Chicago, IL, United States; Fiammetta Soggiu, Royal Free Hospital London, UK; Georg Lurje, University Hospital Aachen, Germany; Gregor A. Stavrou, Klinikum Saarbruecken, Germany; Ioannis Passas, Metropolitan Hospital, Athens, Greece; James Pape, Johannesburg, South Africa; Marc Bemelmans​, Maastricht​ University Medical Center​, Netherlands; Marieke de Boer, University Medical Center Groningen​, Netherlands; Omid​ Madadi-Sanjani​, Royal Free Hospital London, UK; Pim Olthof, University of Amsterdam, Netherlands; Rahul Koti, Royal Free Hospital, UK; Stefen Gilg Karolinska Institutet, Stockholm, Sweden; Steven Olde Damink, Maastricht University, Netherlands; Sven Lang, University Hospital Freiburg, Germany; Theodora Pissanou, Royal Free Hospital London, UK; Thomas Hanna, Royal Free Hospital London, UK; Victoria Ardiles, Hospital Italiano de Buenos Aires, Argentina.

**Country Leaders:**

**Algeria**: Ahmed Belhadj Mostefa, Centre Hospitalo-Universitaire Constantine; **Argentina**: Jeremias Goransky, Hospital Italiano de Buenos Aires; Lucas McCormack, Hospital Aleman of Buenos Aires; Nicolas Resio, Unidad HPB Sur; **Australia**: George Petrou, Mid North Coast; Thomas Hugh, Royal North Shore Hospital; Vincent Lam, Norwest Private Hospital; **Austria**: Thomas Gruenberger, Social Medical Center South; **Belarus**: Aliaksei Shcherba, Minsk Medical Center for Surgery, Transplantation and Hematology; **Belgium**: Claude Bertrand, CHU UCL Namur, Godinne; Mathieu D'Hondt, Groeninge Hospital Kortrijk; **Brazil**: Claudemiro Quireze Junior, Universidade Federal de Goias; Lucio Lucas Pereira, Hospital Sirio Libanes. Felipe José Fernández Coimbra, AC Camargo Cancer Center. **Bulgaria**: Daniel Kostov, Military Medical Academy, Varna; Nikola Vladov, Military Medical Academy, Sofia; Nikolay Belev, UMBAL-Eurohospital; **Canada**: Gonzalo Sapisochin, UHN - Toronto General Hospital; **Chile**: Camila Hidalgo, Nicolas Jarufe, P. Universidad Carolica de Chile; **China**: An'an Gong, Yiwu Central Hospital; Chao Liu, Sun Yat-sen Memorial Hospital, Sun Yat-sen University; Dachen Zhou, The Second Hospital of Anhui Medical University; Yiming Chen, The First Affiliated Hospital of Dali University; Fei Zhang, The First Affiliated Hospital of China Medical University, Zhang Wen, The first affiliated Hospital, Guangxi Medical University; **Croatia**: Mario Kopljar, University Hospital Center "Sestre milosrdnice"; **Cyprus**: Athanasios Petrou, American Institute of Minimal Invasive Surgery; **Czech Republic**: Vladislav Treska, University Hospital; **Egypt**: Ahmed Sherif, National Liver Institute - Menoufia University; Mahmoud El-Meteini, Faculty of Medicine, Ain Shams University; **France**: David Fuks, Institut Mutualiste Montsouris; Olivia Sgarbura, Cancer Institute Montpellier; **Germany**: Bettina M. Buchholz, University Medical Center Hamburg-Eppendorf; Jun Li, University Medical Center Hamburg-Eppendorf; Stefan Heinrich, University Hospital of Mainz; **Greece**: George Tzimas, Hygeia Hospital; Georgios Tsoulfas, Aristotle University of Thessaloniki; Ioannis Passas, Metropolitan Hospital; Panagiotis Petras, Ippokratio General Hospital of Thessaloniki; **India**: Soumil Vyas, Sir H N Reliance Foundation Hospital; **Indonesia**: Budi Irwan, RSUP H. Adam Malik; Erik Prabowo, Kariadi General Hospital / Diponegoro University; **Ireland**: Tom Gallagher, St. Vincent's University Hospital; **Israel**: Riad Haddad, Carmel Medical Center; **Italy**: Adelmo Antonucci, Policlinico of Monza; Elio Jovine, Sant’Orsola University hospital IRCCS; Francesca Ratti, San Raffaele Hospital; Francesco Saverio Papadia, Ospedale Policlinico San Martino; Marcello Maestri, Fondazione IRCCS Policlinico San Matteo, Pavia; **Latvia**: Arturs Ozolins, Pauls Stradins Clinical University Hospital; **Lebanon**: Mohamad Khalife, American University of Beirut; **Libya**: Muhammed Elhadi, University of Tripoli; **Lithuania**: Audrius Dulskas, National Cancer Institute; Marius Paskonis, Vilnius University hospital Santaros Klinikos; Tomas Vanagas, Lithuanian University of Health Sciences; **Malaysia**: Peng Soon Koh, University of Malaya; **Maldives**: Shahi Ghani, Tree Top Hospital; **Mexico**: Alejandro Ramirez Del Val, National Institute of Medical Science and Nutrition Salvador Zubiran; Alejandro Eduardo Padilla Rosciano, National Cancer Institute; Carlos Florez Zorrilla, Centro Médico Nacional 20 de noviembre; Javier Melchor-Ruan, Instituto Nacional de Cancerología; **Netherlands**: Carlijn Buis, UMCG; Marc Bemelmans, Maastricht University Medical Center+; **New Zealand**: Jonathan Koea, North Shore Hospital; **Nigeria**: Bose Ojo-Williams, Olusegun Alatise, Obafemi Awolowo University Teaching Hospitals Complex; **Norway**: Sheraz Yaqub, Oslo University Hospital; **Peru**: Eduardo Anchante Castillo, G. Almenara I. National Hospital Essalud; Peru: Victor Hugo Torres Cuevs, G. Almenara I. National Hospital Essalud; **Philippines**: Catherine Teh, St Luke's Medical Center; **Poland**: Andrzej Komorowski, University of Rzeszow; Krzysztof Jeziorski, National Institute of Geriatrics, Rheumatology and Rehabilitation, Warsaw; Krzysztof Zieniewicz, Medical University of Warsaw; Oskar Kornasiewicz, Medical University of Warsaw; **Portugal**: Vitor Nunes, Hospital Prof. Dr. Fernando Fonseca; **Qatar**: Hatem Khalaf, Hamad Medical Corporation; **Romania**: Anamaria Schipor, Iuliu Haţieganu University of Medicine and Pharmacy; Ionut Negoi, Emergency Hospital of Bucharest, Carol Davila University of Medicine and Pharmacy Bucharest; Matei Bratu, Emergency Clinical Hospital of Bucharest; Octav Ginghina, Saint John Hospital Bucharest; **Russia**: Arkady Bedzhanyan, Russian Reserch Center of Surgery named after B.V.Petrovsky; Ivan Kozyrin, Clinical Hospital #1 MEDSI; Nikita Chardarov, Petrovsky Russian Research Center of Surgery; Nikolay Bagmet, Petrovsky National Research Centre of Surgery; Vladimir Zagainov, Volga District Medical Center of FMBA of Russia; **Saudi Arabia**: Ahmed Zidan, King Faisal Specialist Hospital and Research Centre; **Serbia**: Aleksandar Karamarkovic, Clinic for Surgery, University Clinical Center Zvezdara, Faculty of Medicine University of Belgrade; Daniel Galun, University Clinic for Digestive Surgery; Mladjan Protic, University of Novi Sad, Faculty of Medicine; **Slovakia**: Alexander Ferko, University Hospital Martin; **Slovenia**: Blaž Trotovšek, University Medical Centre Ljubljana; Slovenia: Peter Pipan, TBA; **Spain**: Benedetto Ielpo, University Hospital of Leon; **Spain**: Irene Ortega, Infanta Sofía University Hospital; JM Asencio, Hospital General Universitario Gregorio Marañón; Miguel-Angel Suarez-Muñoz, University Hospital "Virgen de la Victoria"; Roberto Brusadin, Virgen del Arrixaca Clinic and University Hospital; Vicenç Artigas, Universidad Autònoma Barcelona; **Sri Lanka**: Bulathsinhalage Bulathsinhala, Colombo North Teaching Hospital;Rohan Siriwardana, University of Kelaniya; **Sweden**: Ernesto Sparrelid, Karolinska Institutet; Per Sandström, Institution of Biomedical and Clinical Sciences; **Switzerland**: Andrea Peloso, HUG - Hôpitaux Universitaires de Genève; Erik Schadde, Cantonal Hospital Winterthur; **Turkey**: Ahmet Coker, Ender Dulundu, University of Istanbul, Cerrahpasa, School of Medicine; Mustafa Kerem, Gazi University Faculty of Medicine; **United Kingdom**: Francis Robertson, Royal Infirmary of Edinburgh; Moh'd Abu Hilal, Southampton University Hospital and Poliambulanza Foundation Hospital, Brescia, Italy; Rajiv Lahiri, Royal Free Hospital; Reena Ravikumar, Kings College Hospital; Robert Hutchins, Bart's Health NHS trust; Satheesh Iype, Royal Free Hospital; Stephanos Pericleous, The Royal Marsden NHS Trust; VM Bobby Dasari, Queen Elizabeth Hospital; **United States:** Alan Koffron, Beaumont Health System; Emmanouil Giorgakis, University of Arkansas for Medical Sciences; Hrishikesh Samant, Willis Knighton health system; Steven Curley, Christus Trinity Mother Frances. Yuri Genyk, University of Southern California, **Uruguay**: Daniel Czarnevicz, Servicio Medico Integral.

**LiverGroup.org Members:**

**Argentina**: **HIGA Dr. O. Alende Hospital**: Gabriela Delvalle; **Hospital Aleman of Buenos Aires**: Matias Balmer; **Hospital Universitario Fundacion Favaloro**: Diego Ramich; Gabriel Gondolesi; Pablo Barros Schelotto; **Clinica Pasteur Rio Negro**: Nicolas Resio; **Unidad HPB Sur**: Dario Abaca; Julio Lazarte; **Australia**: **Norwest Private Hospital**: Lawrence Yuen; Tony Pang; **Royal North Shore Hospital**: Nazim Bhimani; **Austria: Community Hospital Horn**: Andreas Hauer; Klaus Kirbes; Reinhold Klug; **Medical University of Innsbruck**: Eva Braunwarth; Florian Primavesi; Stefan Stättner; **Belarus**: **Minsk Medical Center for Surgery, Transplantation and Hematology**: Dmitry Fedaruk; Sergey Korotkov; **Belgium**: **AZ Delta**: Sébastien Strypstein; Bart Smet; Mehrdad Biglari; **CHU UCL Namur, Godinne**: Alexandra Dili; **Ghent University Hospital**: Aude Vanlander; Betsy Van Loo; Luís Filipe Abreu de Carvalho; Bart Hendrikx; Federico Tomassini; Xavier Rogiers; **Groeninge Hospital Kortrijk**: Celine De Meyere; Franky Vansteenkiste; **Brazil**: **Santa Casa de Misericórdia do Pará**: Fernanda Oliveira Barreto Garcia; Rafael José Romero Garcia; **Universidade Federal do Pampa /Hospital Santa Casa de Caridade de Ururguaiana:** Diego Kleinubing; **Universidade Federal de São Paulo, Hospital São Paulo**: Marcelo Moura Linhares; Leandro Dias Cezar; Daniel Kitayama Shiraiwa; Rachel Riera; **Bulgaria**: **Military Medical Academy**: Tsonka Lukanova; Vasil Kostov; **UMBAL-Eurohospital**: Panche Krastev; Radoslav Penkov; **Chile: P. Universidad Carolica de Chile**: Martin Dib; P. Universidad Católica de Chile: Carlo Marino; **China: The Second Hospital of Anhui Medical University:** Dachen Zhou, Jiong Gu, Bin Zhang; **Yat-sen Memorial Hospital, Sun Yat-sen University**: Lei-bo Xu; Rui Zhang; **The First Affiliated Hospital of Dali University**: Yunbo Tan; Ziting Su; **The first affiliated Hospital, Guangxi Medical University**: Banghao Xu; Jilong Wang; **Yiwu Central Hospital**: Ke Miao; Xuhang Luo; **Yuebei Peopl’s Hospital**: Haimin Chen; Jiafeng Zhao; **Croatia**: **University Hospital Center "Sestre milosrdnice"**: Mario Zovak; **Czech Republic: University Hospital**: Jakub Fichtl; **Egypt**: **Faculty of Medicine, Ain Shams University**: Hany Dabbous; Mohamed Bahaa; **National Liver Institute - Menoufia University**: Islam Ayoub; Maher Osman; **Estonia**: **University of Tartu: Jaan Soplepmann**; Margus Kivisild; Olav Tammik; **France**: Cancer Institute Montpellier: François Quenet; **Centre Hospitalier Régional Universitaire de Lille**: Stéphanie Truant; Emmanuel Boleslawski; **Hôpital Ambroise Paré - APHP:** Renato M Lupinacci; Frédérique S Peschaud; **Institut Mutualiste Montsouris:** Ela Ekmekçigil; Ioannis Triantafyllidis; **Germany: Asklepios Clinic Barmbek**: Georgios Makridis; Alexandros Kantas; Tim Reese; **Heinrich Heine University Hospital:** Nadja Lehwald-Tywuschik; Paulina Shabes; Wolfram Knoefel; **Medical Center Freiburg**: Magdalena Menzel; **Saarbruecken Hospital**: Dimitrios Kardassis; **Technical University Munich:** Christian Stöß; Daniel Hartmann; Alexander Novotny; Helmut Friess; **University Hospital Heidelberg:** Arianeb Mehrabi; **University Hospital of Mainz**: Hauke Lang; Verena Tripke; **University Hospital Regensburg**: Hans Jürgen Schlitt; Jens M. Werner; Monika Diehl-Bein; Stefan M. Brunner; **University Hospital RWTH Aachen**: Isabella Lurje; Zoltan Czigany; **Greece**: **Aretaieion Hospital, National and Kapodistrian University Medical School of Athents**: Konstantinos Bramis; Manousos Konstadoulakis; **Hippokration General Hospital, National and Kapodistrian University Medical School of Athents:** Nikolaos Alexakis; **Aristotle University of Thessaloniki:** Dimitrios Giakoustidis; Vasileios Papadopoulos; **Attikon University Hospital:** Anna Paspala; Emmanuel Pikoulis; Nikolaos Arkadopoulos; Panagiotis Kokoropoulos; Paul Patapis; Theodoros Sidiropoulos; **Evgenideio Hospital:** Nikolaos Machairas; Paraskevas Stamopoulos; **Hygeia Hospital**: George Tzimas; Spiridon Pangratis, **Ippokratio General Hospital of Thessaloniki:** Apostolos Kambaroudis; Thomas Kanteres; **Laiko General Hospital:** Dimitrios Dimitroulis; Georgios Sotiropoulos; Michail Vailas; Zoe Garoufalia; **Laiko General Hospital, Athens Medical School**: Alexandros Papalampros; John Griniatsos; **Metropolitan General**: Emmanuil Zacharakis; Theo Tsirlis; Ioannis Sideris; **National and Kapodistrian University of Athens:** Athanasios Syllaios; Dimitrios Schizas; Konstantinos Toutouzas; Natasha Hasemaki; **Olympion General Clinic:** Apollon Zygomalas; Dionissios Karavias; Nikolaos Katsiakis; **Saint Savvas Anticancer Hospital:** Dimitrios Balalis; Dimitrios Korkolis; Dimitrios Manatakis; **University Hospital of Ioannina and School of Medicine of Ioannina:** Ioannis Kyrochristos; Georgios Glantzounis; **University Hospital of Larissa:** Alexandros Diamantis; Konstantinos Perivoliotis; Konstantinos Tepetes; **University of Athens:** Evangelos Felekouras; Dimitrios Moris; **India**: **Amrita Institute of Medical Sciences**: Biju Chandran; Christi Varghese; Surendran Sudhindran; **Caritas Hospital**: Arun Kumar; Murali Appukuttan; Vinitha Nair; **Hindu Mission Hospital, Chennai:** Anand Ramamurthy; **Shalby Hospitals**: Bhavin Vasavada; Hardik Patel; **Indonesia**: **M.djamil Padang General Hospital**: Irwan Rachman; **Prof. Dr. R.D. Kandou General Hospital**: Michael Tendean; **Ireland**: **St. Vincent's University Hospital**: Aidan O'Dowling; Emir Hoti; Patrick Kambakamba; **Israel**: **Carmel Medical Center**: Moneer Swaed; Riad Haddad; **Rabin Medical Center:** Eran Sadot; Hanoch Kashtan; **Rambam Health Care Campus:** Offir Ben-Ishay; Safi Khouri; **Italy**: **AOUI Verona**: Luca Bortolasi; **ASST Papa Giovanni XXIII, Bergamo**: Carolina Rubicondo; Mara Giovanelli; AUSL Bologna: Matteo Zanello; Michele Masetti; Raffaele Lombardi; **Azienda Ospedaliera "Vito Fazzi":** Marcello Spampinato; Stefano Garritano; **Azienda Ospedaliera Papardo:** Edoardo Saladino; Giuseppe Cuticone; Nino Gullà; **Dell'Angelo Hospital:** Alfonso Recordare; Fabrizio Cimino; **Fondazione IRCCS Istituto Nazionale dei Tumori:** Alessandro Germini; Vincenzo Mazzaferro; Claudia Piscitelli; Tommaso Dominioni; **AOUI Verona:** Alfredo Guglielmi; Andrea Ruzzenente; Tommaso Campagnaro; **IRCCS - Regina Elena National Cancer Institute:** Chiara Parrino; Gian Luca Grazi; Valerio De Peppo; **La Maddalena:** Marco Paci; Lucio Mandalà; Pietro Mezzatesta; **Mauriziano Hospital:** Alessandro Ferrero; Fabio Forchino; Nadia Russolillo; **National Cancer Institute - Fondazione "G. Pascale" - IRCCS:** Andrea Belli; Vincenza Granata; Francesco Izzo; **Ospedale Centrale di Bolzano**: Antonio Frena; Stefan Patauner; Giovanni Scotton; Francesca Notte; **Ospedale Policlinico San Martino:** Andrea Massobrio; Davide Pertile; Franco De Cian; Stefano Di Domenico; Stefano Scabini; **Policlinico di Monza:** Marco Mattioli; Ana Gonta; Sciannamea Ivano; **Policlinico S.Orsola-Malpighi**: Matteo Serenari; **Policlinico San Pietro:** Enrico Pinotti; Mauro Montuori; Mauro Zago; **San Raffaele Hospital:** Federica Cipriani; Luca Aldrighetti; **Sant'Orsola Hospital:** Margherita Binetti; Maurizio Cervellera; Valeria Tonini; **Università Politecnica delle Marche:** Federico Mocchegiani; Grazia Conte; Marco Vivarelli; **University of Milan-Bicocca:** Alessandro Giani; Fabrizio Romano; Simone Famularo; **University of Sassari:** Alberto Porcu; Claudio F. Feo; Giuseppe Cherchi; Lebanon: American University of Beirut: Walid Faraj; Libya: Alkhadra Hospital: Bushray Almiqlash; **Libya**: **Tripoli University Hosptial**: Hazem Ahmed; **Zliten Teaching Hospital**: Ahmad Alzedam; **Lithuania**: **National Cancer Institute:** Eugenijus Stratilatovas; Rimantas Bausys; **Vilnius University hospital Santaros Klinikos**: Kestutis Strupas; Rokas Rackauskas; **Lithuanian University of Health Sciences:** Giedrius Barauskas; Romualdas Riauka; **Malaysia**: **University of Malaya**: Boon Koon Yoong; Jun Kit Koong; **Mexico**: National Cancer Institute: Horacio Lopez Basave; **National Institute of Medical Science and Nutrition Salvador Zubiran**: Alan Contreras Saldívar; Ismael Dominguez Rosado; Mario Vilatobá Chapa; Miguel Mercado; **Netherlands**: **Albert Schweitzer Hospital**: Eric Belt; Gijs Musters; Joost Van der Hoeven; **Amphia Ziekenhuis:** Arjen Rijken; Jan Wijsman; Paul Gobardhan; **Amsterdam University Medical Center**: Joris Erdmann; Marc Besselink; Rutger-Jan Swijnenburg; **Antoni van Leeuwenhoek Ziekenhuis**: Koert Kuhlmann; Niels Kok; Theo Ruers; **Deventer Ziekenhuis**: A. Talsma;

Hans Torrenga; Stijn Vanlaarhoven; **Dutch Institute for Clinical Auditing / UMCG:** Arthur Elfrink; **Erasmus Medical Center**: Dirk Grünhagen; C. Verhoef; Michiel Rothbarth; **Gelre Ziekenhuis:** Peter van Duijvendijk; **IJsselland Ziekenhuis:** Elisabeth de Wijkerslooth; Maarten Vermaas; Pascal Doornebosch; Isala Klinieken: Gijs Patijn; V. Nieuwenhuis; **Maastricht University Hospital:** Marcel den Dulk; **Maastricht University Medical Center+:** Kees Dejong; Marielle Coolsen; **Maxima Medical Center:** Gerrit Slooter; Wouter Leclerq; Julie Sijmons; **Medical Center Leeuwarden:** Christiaan Hoff; Eric Manusama; Hasan Eker; **Medisch Spectrum Twente:** Daan Lips; Jorieke Nijhuis; Mike Liem; **Onze Lieve Vrouwe Gasthuis:** Fadi Rassam; Hendrik Marsman; Michael Gerhards; **Radboud University Medical Center:** Peter van den Boezem; Johannes de Wilt; Martijn Stommel; **Regionaal Academisch Kankercentrum Utrecht:** Jeroen Hagendoorn; Wouter te Riele; **Spaarne Gasthuis:** Ronald Vuylsteke; Steven Oosterling; **University Medical Centre Groningen:** Frederik Hoogwater; Marieke de Boer; Floris Poelmann; **Deventer Ziekenhuis:** H. Torrenga; K. Talsma, **New Zealand: North Shore Hospital:** Michael Rodgers; Universe Leung; **Auckland City Hospital:** Peter Johnston; John McCall; **Waikato Hospital:** Fraser Welch; **Christchurch Hospital:** Saxon Connor; Todd Hore; **Dunedin Hospital:** Andrew Audeau; **Peru**: **G. Almenara I. National Hospital Essalud:** Lilian Rebecs Mantilla Silva; **Philippines**: **St Luke's Medical Center**: Amornetta Jordan-Casupang; **Poland**: **Medical University of Warsaw:** Bartosz Cieslak; Bartosz Maczkowski; Dariusz Wasiak; Maciej Kosieradzki; Maciej Krasnodębski; Marcin Kotulski; Michał Grąt; Piotr Kalinowski; Piotr Malkowski; Piotr Smoter; Rafał Paluszkiewicz; Waclaw Hołówko; Waldemar Patkowski; **Portugal**: **Centro Hospitalar Universitario Sao Joao - Porto:** Renato de Melo; **Hospital Prof. Dr. Fernando Fonseca:** Antonio Gomes; Rui Marinho; **Qatar**: **Hamad Medical Corporation:** Ahmed Elaffandi; Walid El-Moghazy; **Republic of Korea: Seoul National University Bundang Hospital, Seoul National University College of Medicine**: Boram Lee, Jai Young Cho, Ho-Seong Han **Reunion**: **CHU Réunion**: Herve Fagot; Johanna Zemour; **Romania**: **Emergency Clinical Hospital of Bucharest**: Bogdan Diaconescu; Bogdan Stoica; Mircea Beuran, Valentina Madalina Negoita, Cezar Ciubotaru;; Saint John Hospital Bucharest: Andrada Spanu; Mara Mardare; **Russia**: **Petrovsky National Research Centre of Surgery:** Garnik Shatverian; Nikolay Bagmet; Konstantin Petrenko; Lilia Polishchuk; **Volga District Medical Center of FMBA of Russia**: Gleb Gorochov; Nikolay Bobrov; **Saudi Arabia**: **King Faisal Specialist Hospital and Research Centre**: Mark Sturdevant; **Serbia**: **Clinic for Surgery, University Clinical Center Zvezdara, Faculty of Medicine University of Belgrade**: Jovan Juloski; Ljiljana Milic; Vladica Ćuk**. University Clinic for Digestive Surgery:** Aleksandar Bogdanovic; Marko Zivanovic; **University of Novi Sad, Faculty of Medicine**, Oncology Institute of Vojvodina; **Slovakia: University Hospital Martin:** Ludovit Laca; Martin Vojtko; **Slovenia**: **University Medical Centre Ljubljana:** Miha Petrič; Mihajlo Djokić; **Spain**: **Hospital Universitari de Girona Dr Josep Trueta:** Maria Teresa Albiol; Ernest Castro; Santiago López-Ben; **Hospital Torrecardenas:** Maria del Mar Rico; Miguel Vargas; Orlando Porcel; **Hospital Universitari Son Espases:** Francesc Xavier Molina Romero; José Miguel Morón Canis; Natalia Pujol Cano; **Hospital Universitario Central de Asturias:** Alberto Miyar de León; Carmen García Bernardo; Lorena Solar-García; **Hospital Universitario de Badajoz:** Adela Rojas Holguín; Diego López Guerra; Gerardo Blanco Fernández; **Hospital Universitario de Canarias:** Ángel Pallares; Antonio Martín Malagón; Liliana Pezzetta Hernández; **Hospital Universitario de Guadalajara**: Alba Manuel; Jose Ramia; Raquel Latorre; **Hospital Universitario de Torrejón**: Enrique Esteban Agustí; Maria Gutiérrez Samaniego; Miguel-Angel Hernández Bartolomé; **Hospital Universitario de Valme:** Darío Martinez-Baena; José Lorente-Herce; Pablo Parra-Memrbives; **Hospital Universitario Fundación Jiménez Díaz:** Ángel Uriarte; Santiago Ayora; **Hospital Universitario Insular de Gran Canaria:** José López Fernández; Gabriel Garcia Plaza; Javier Alcalá Serrano; **Hospital Universitario Puerta del Mar:** Maria Jesús Castro Santiago; **Infanta Sofía University Hospital**: Ángel Cuadrado; Rocío Fernández; **La Paz Hospital:** Jose Castell; Nuria Boñar; Rula Sabbagh; **Miguel Servet University Hospital Zaragoza:** Mario Serradilla; Sandra Paterna; Teresa Gimenez-Maurel; **Hospital Universitario del Mar:** Fernando Burdío; Patricia Sánchez-Velázquez;: Manuel Rodriguez Blanco; **Universidad Autònoma Barcelona**: Manuel Rodriguez Blanco; **Universitario la Fe**: Eva Montalvá; Rafael López-Andújar; **University Hospital "Virgen de la Victoria":** Jorge Roldan de la Rua; Luis Hinojosa-Arco; **University Hospital La Princesa**: Elena Martin-Perez; Marcello Di Martino; Ángela de la Hoz Rodríguez**. University Hospital San Juan de Alicante:** Antonio F. Compañ Rosique, Rumyana Rumenova Smilevska; **University Hospital Virgen del Rocio:** Francisco Javier Padillo Ruiz; Miguel-Angel Gomez Bravo; Pablo Beltran Miranda; **Virgen del Arrixaca Clinic and University Hospital**: Ricardo Robles Campos; Víctor López-López; **University Hospital of Tarragona Joan XXIII:** Mihai Calin Pavel; Laia Estalella; Robert Memba; Rosa Jorba; **Sri Lanka: University of Kelaniya:** Nattashi Ranaweera; Nisansala Harshani; **Sweden**: **Institution of Biomedical and Clinical Sciences**: Bergthor Bjornsson; Linda Lundgren; **Umeå University**: Oskar Hemmingsson; **Switzerland**: **Hôpitaux Universitaires de Genève:** Nicola Colucci; Christian Toso; **Kantonsspital St.Gallen:** Antonia Loosen; Fariba Abbassi; Thomas Steffen; **Kantonsspital Winterthur**: Erik Schadde; Franziska Heid; **University Hospital of Lausanne CHUV**: Emmanuel Melloul; Ismail Labgaa; Nicolas Demartines; **Régional Hospital Lugano:** Alessandra Cristaudi; Raffaello Roesel; Pietro Majno; **Taiwan**: **National Taiwan University/Fu Jen Catholic University Hospital**: Po-Chih Yang; **Turkey**: **Ankara University**: Elvan Kirimker; Kaan Karayalcin; **EGE University**: Alper Uguz; Omer Unalp; **Medical Park hospital & Sisli Etfal hospital:** Ertan Emek; Pinar Yazici; **Gazi University Faculty of Medicine:** Ali Yalcinkaya; Hasan Bostanci; Kursat Dikmen; **Ukraine**: **National Cancer Institute**: Oleksandr Kvasivka; Kostiantyn Kopchak; Valeriya Sumarokova; Dmitry Cheverdiuk; Oleg Vasilev; Sergei Sikachov; **United Kingdom**: **Kings College Hospital:** Christina Boumpoureka; Diana Bogatu; Dimitra Intzepogazoglou; Parthi Srinivasan; **Manchester Royal infirmary**: Ajith K Siriwardena. **LiverGroup.org Headquarters London:** Eirini Liova; **Plymouth NHS Trust:** Urszula Donigiewicz; **Princess Grace Hospital:** Lisa Woodrow; **Queen Elizabeth Hospital Birmingham:** Andrea Schlegel; Buddhika Dassanayake; **Royal Blackburn Hospital:** Ambareen Kausar; **Royal Free Hospital London:** Andrea Tufo; Adam Framptonl; Alejandro Ramirez Del Var, Andrea Monaco; Dimitri A Raptis; Brian R Davidson; Charles Imber; David Nasralla; Danielle Ferraro; Dinesh Sharma; Giuseppe Kito Fusai; Helen Tzerbinis; Ioannis D. Kostakis; Joao Mestre de Costa; Joerg-Matthias Pollok; Nikolaos Dimitrokallis; Pascale Tinguely; Satheesh Iype, Stephanos Pericleous; Timothy Owen; Theodora Pissanou; **Royal Infirmary of Edinburgh**: Ewen Harrison; **Southampton University Hospital:** John Primrose; Thomas Armstrong; Christoph Kuemmerli; Christoph Tschuor; Raed Aljarrah; **St james University Hospital:** Peter Lodge; Philipp Kron; **The Royal Marsden NHS Trust:** Daniel Akhtar; Tania Policastro; Ricky H Bhogal. **United States**: **Carolinas Medical Center - Atrium Health**: Christoph Tschuor; Dionisios Vrochides; John Martinie; **Rush University Medical Center**: JJ Klein; Erik Schadde; Martin Hertl; Xavier Keutgen; Jennifer Kalil; **University of Arkansas for Medical Sciences:** Lyle Burdine; **University of Rochester Medical Center:** Katie Helbig; **Uruguay**: **Servicio Medico Integral**: Gustavo Andreoli; Santiago Cubas.

## Supplementary Material

SUPPLEMENTARY MATERIAL
