# Supplementary material for: Outcomes of elective liver surgery worldwide: a global, prospective, multicenter, cross-sectional study
Source: Int J Surg. 2023 Oct 4;109(12):3954–66. doi: 10.1097/JS9.0000000000000711 (PMC10720814; doi:10.1097/JS9.0000000000000711)
Supplement: SUPPLEMENTARY MATERIAL [file js9-109-3954-s001.docx]

**Supplementary Figures**


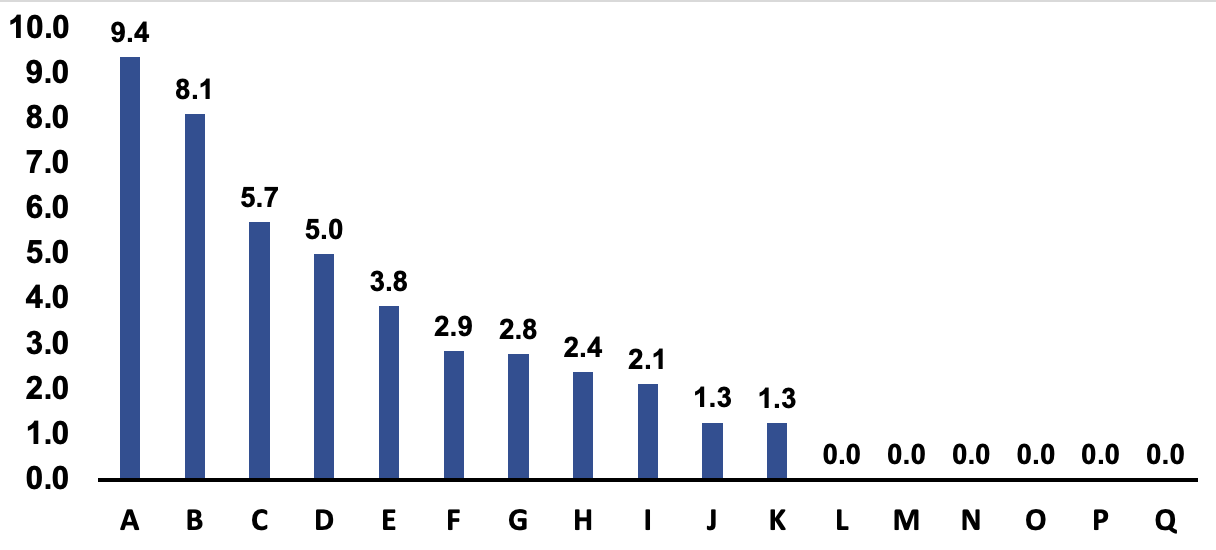


**Supplementary Figure 1.** Mortality rates among the high-volume centers (n>30 cases performed)


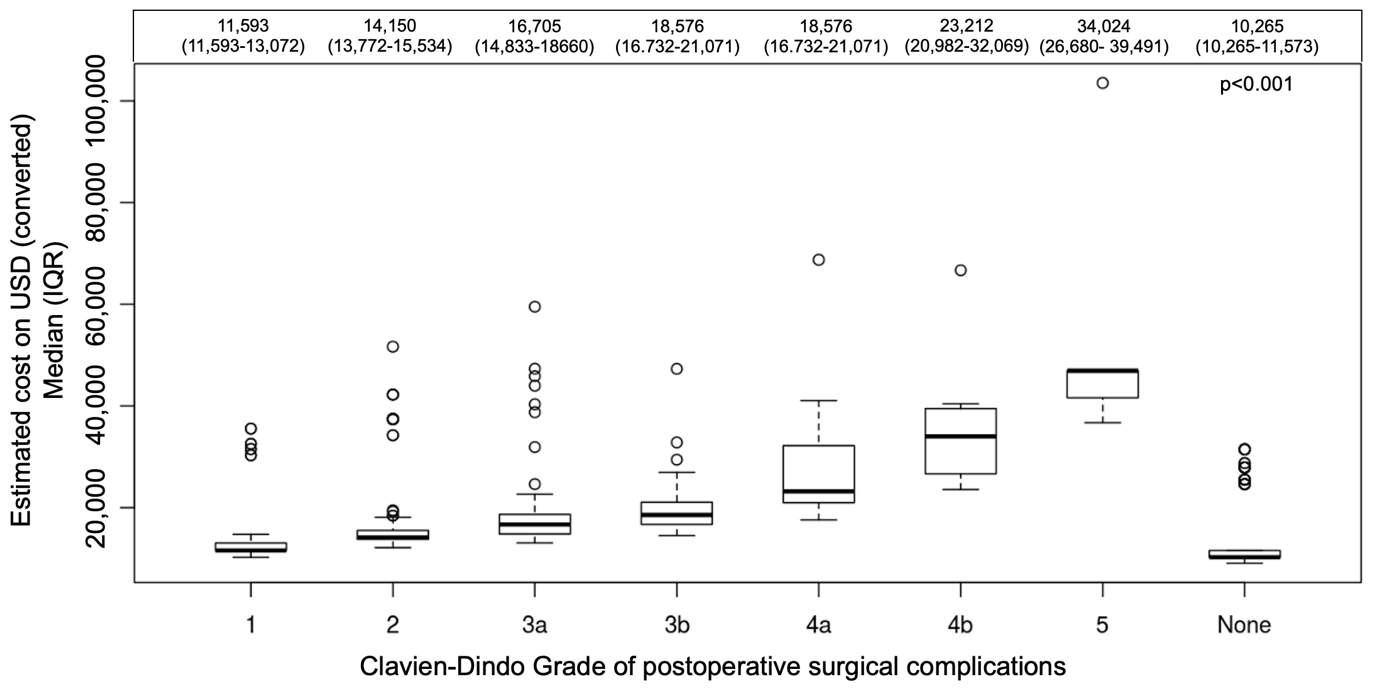


**Supplementary Figure 2.** Costs in USD related to different severities of postoperative complications


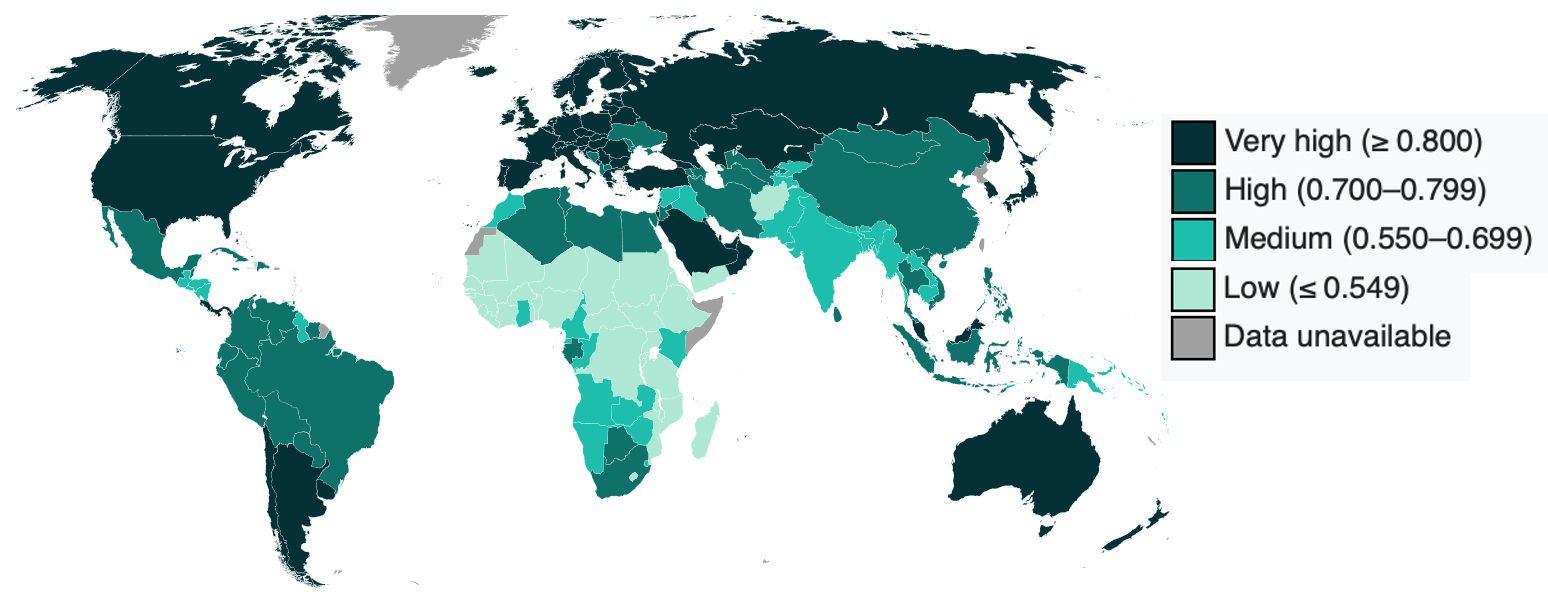


**Supplementary Figure 3.** The Human Development Index (HDI) among all countries worldwide


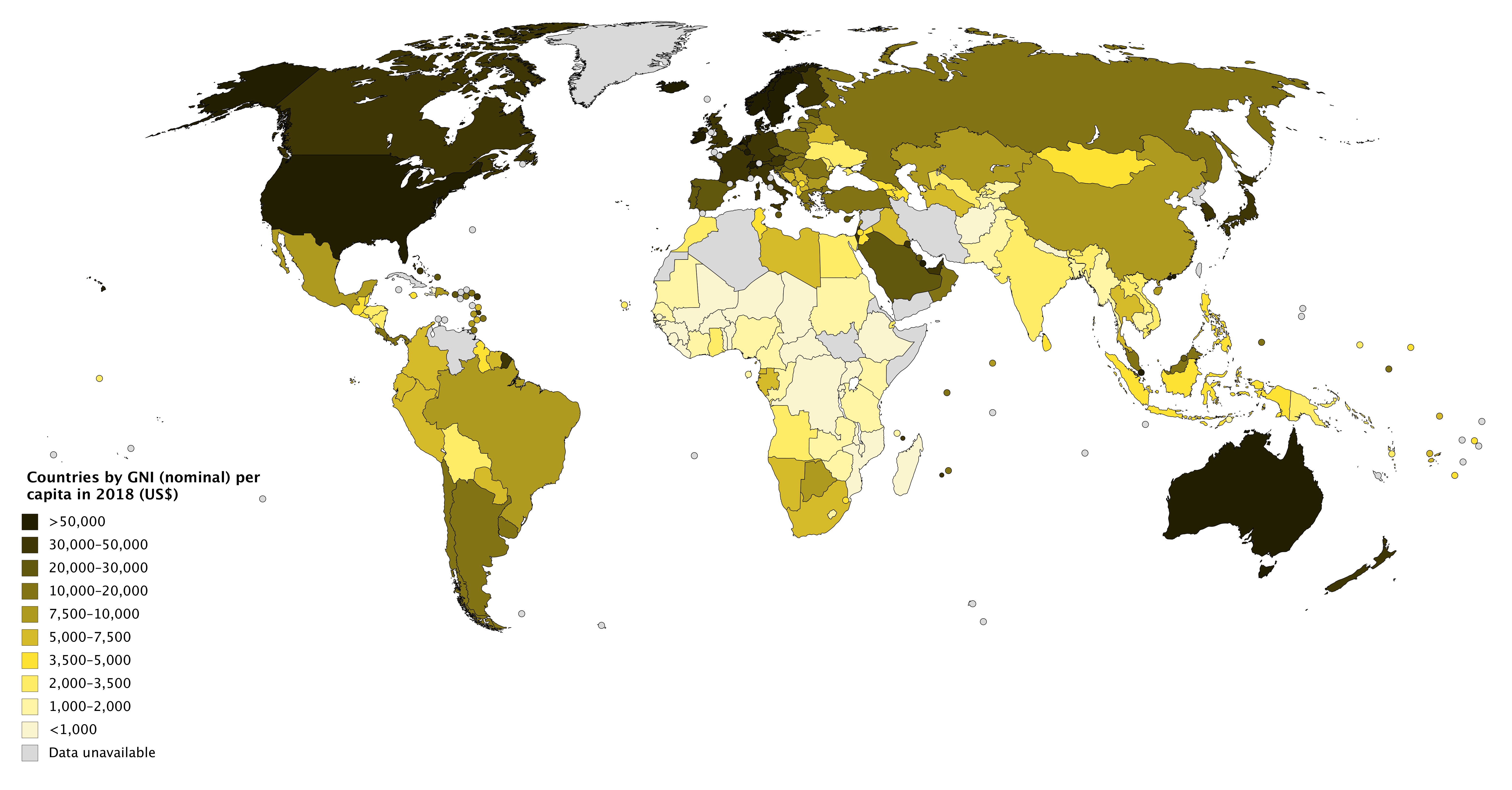


**Supplementary Figure 4.** Gross National Income per capita among countries worldwide


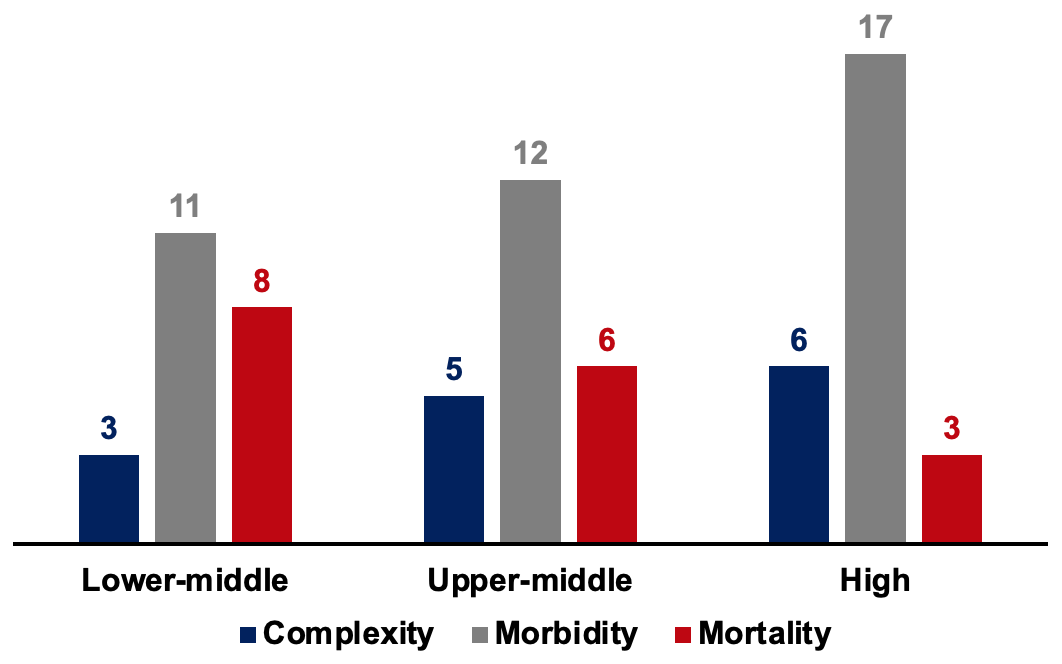


**Supplementary Figure 5.** Complexity of liver surgery, morbidity, and mortality among the groups of Gross National Income (GNI) per capita


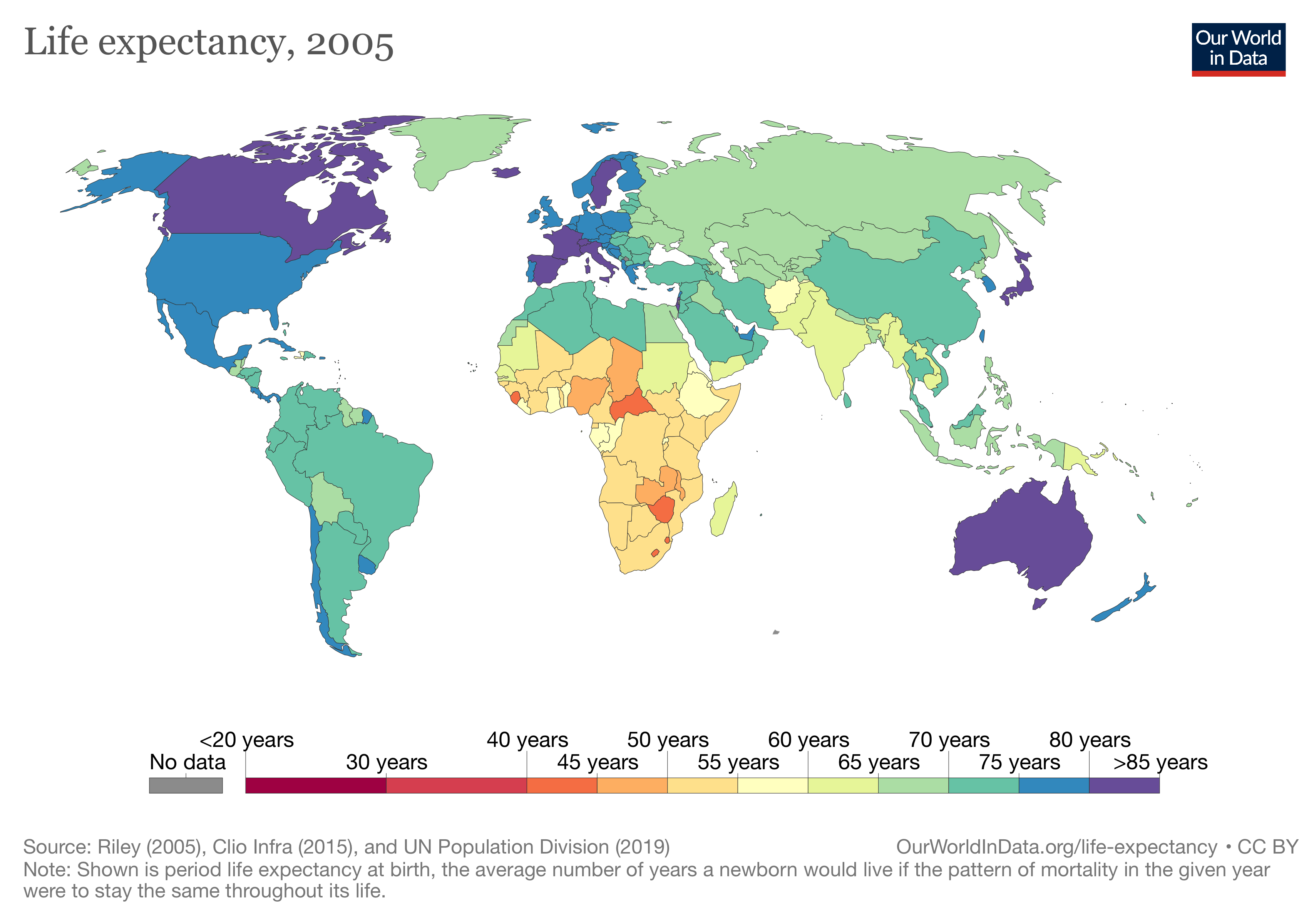


**Supplementary Figure 6.** Life expectancy index among countries worldwide


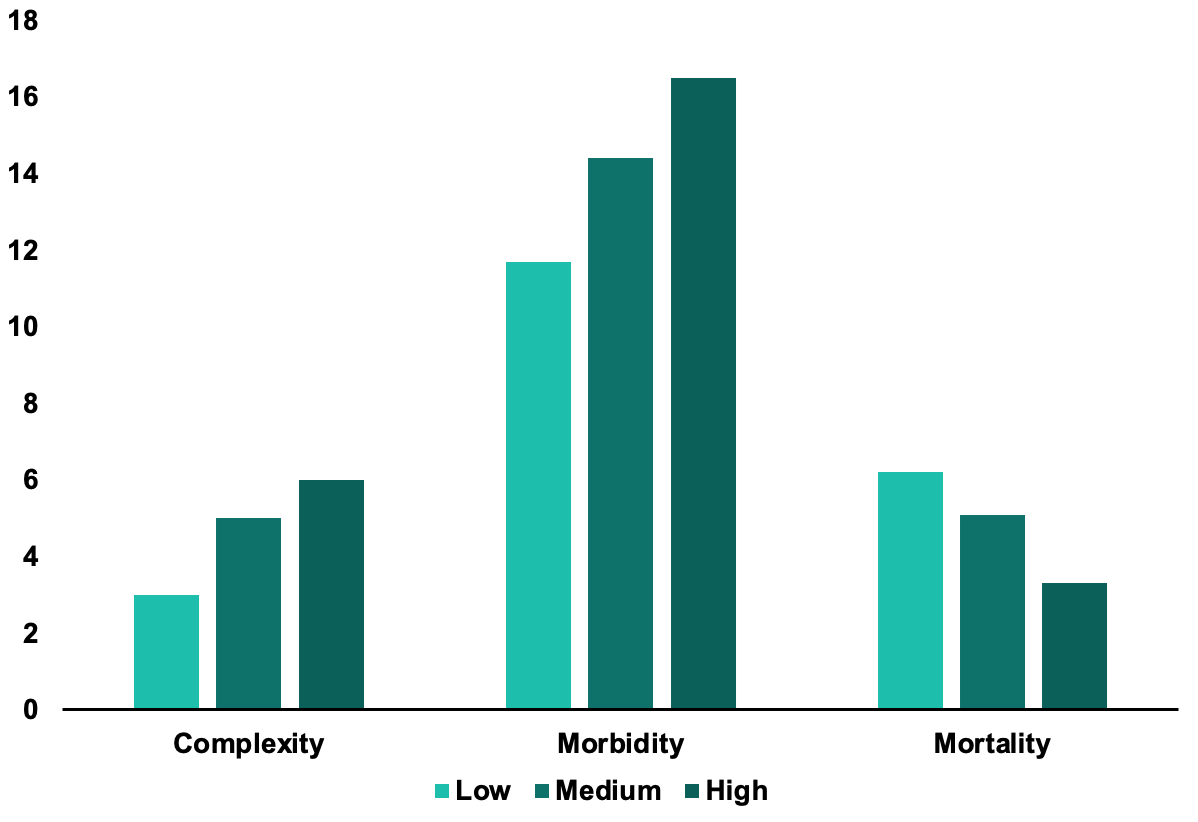


**Supplementary Figure 7.** Complexity of liver surgery, morbidity, and mortality among the groups of Life Expectancy Index


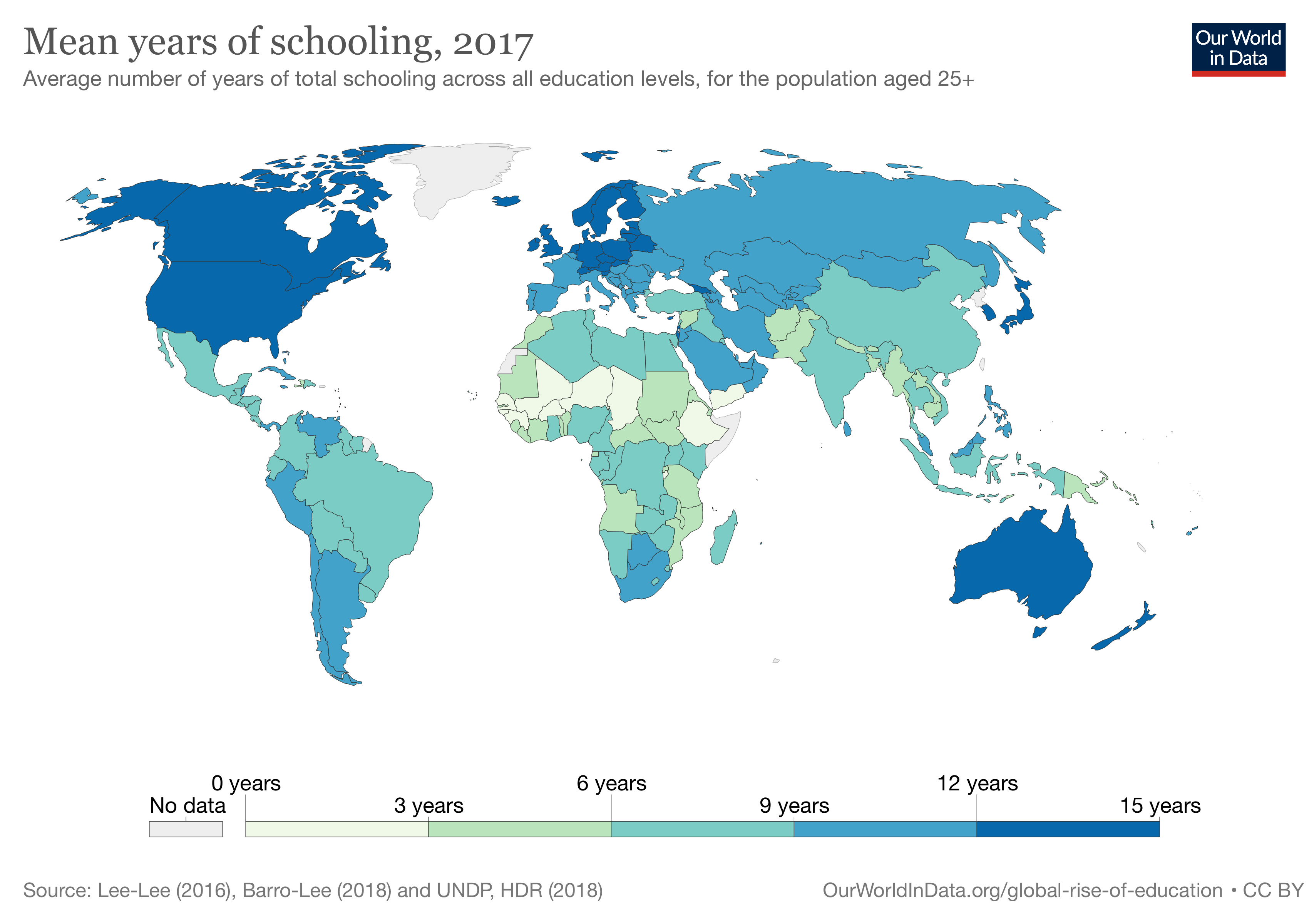


**Supplementary Figure 8.** Mean Years of Schooling among countries worldwide


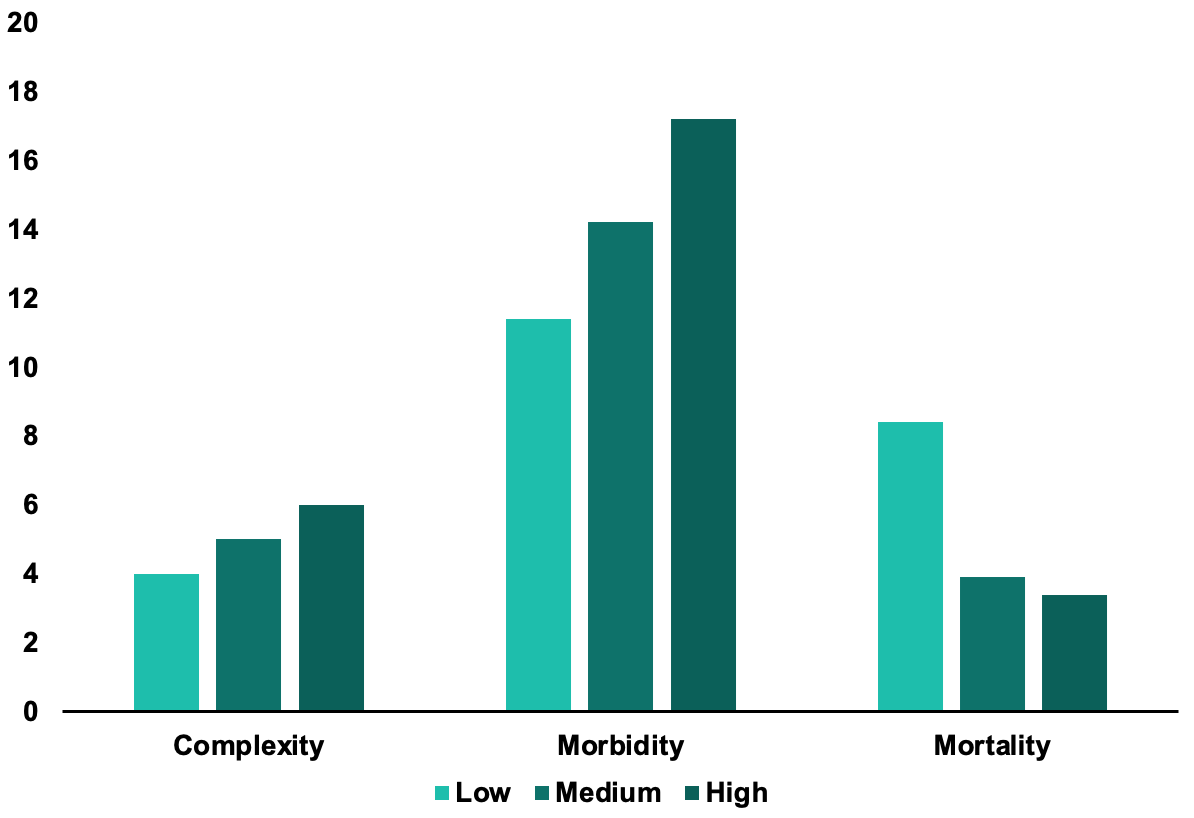


**Supplementary Figure 9.** Complexity of liver surgery, morbidity, and mortality among the groups of Mean Years of Schooling


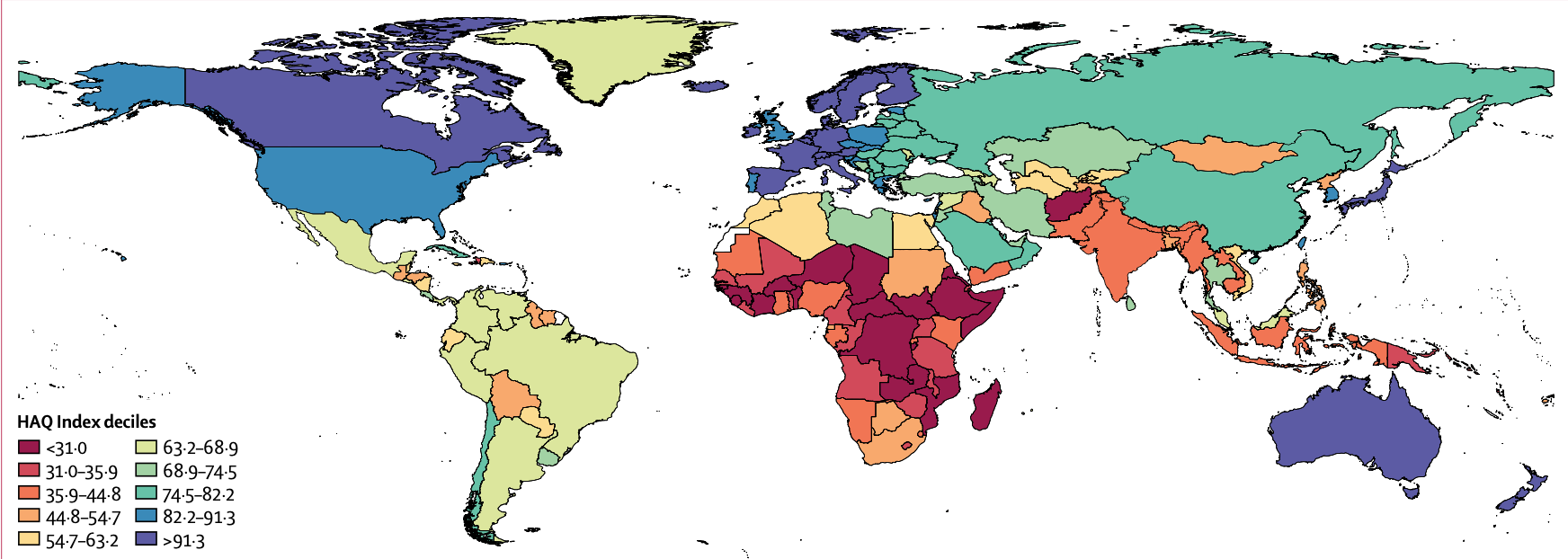


**Supplementary Figure 10.** Health Access and Quality (HAQ) Index among countries worldwide


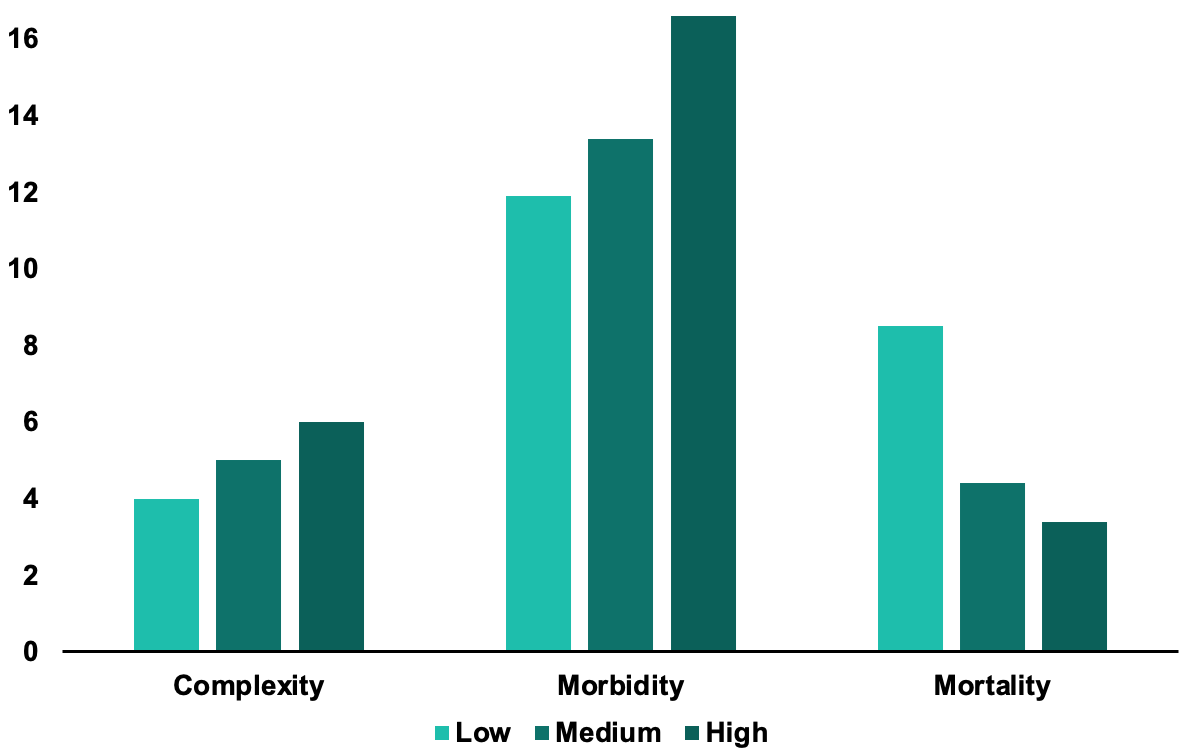


**Supplementary Figure 11.** Complexity of liver surgery, morbidity, and mortality among the groups of Health Access and Quality (HAQ) Index


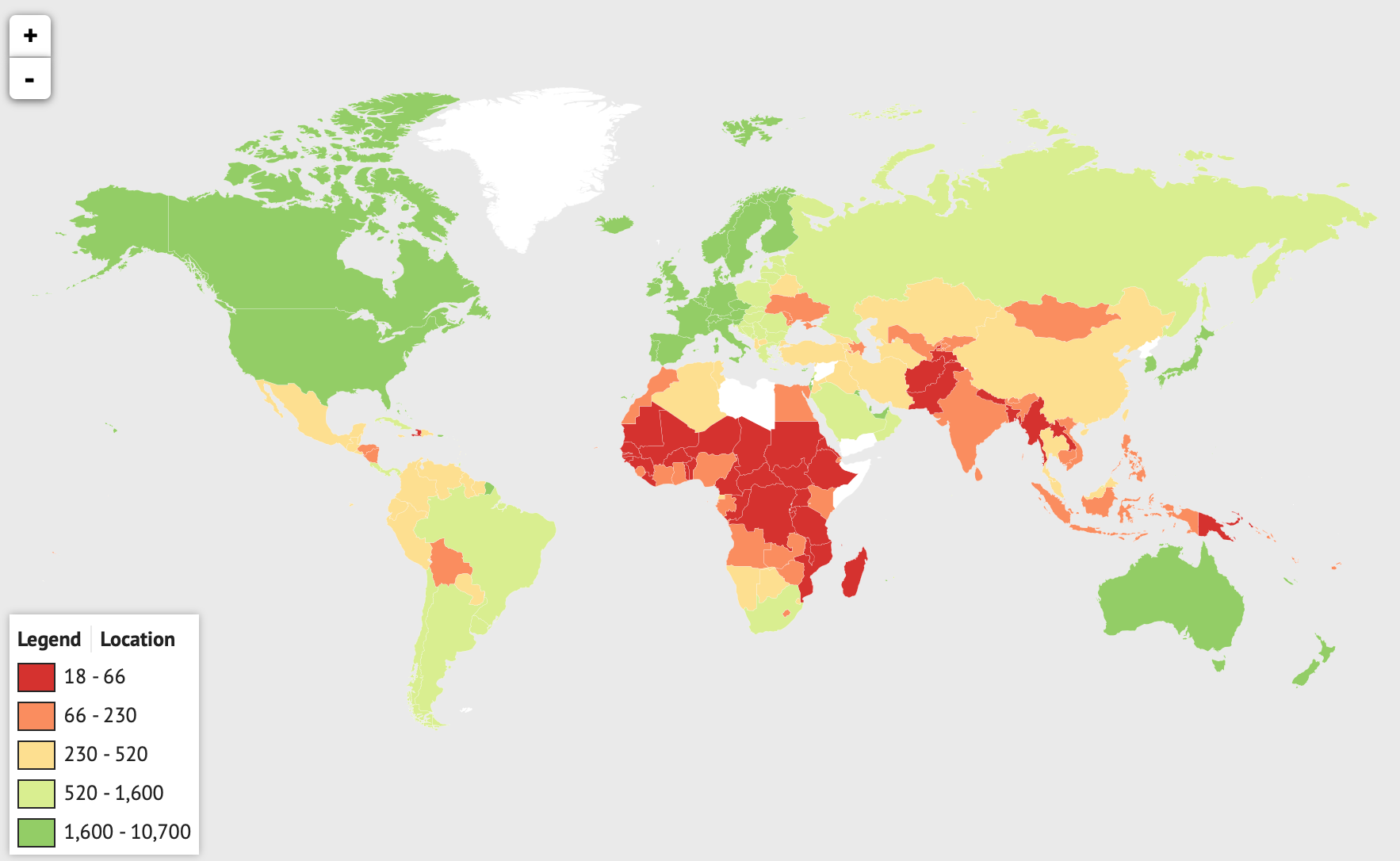


**Supplementary Figure 12.** Total health expenditure per capita among countries worldwide


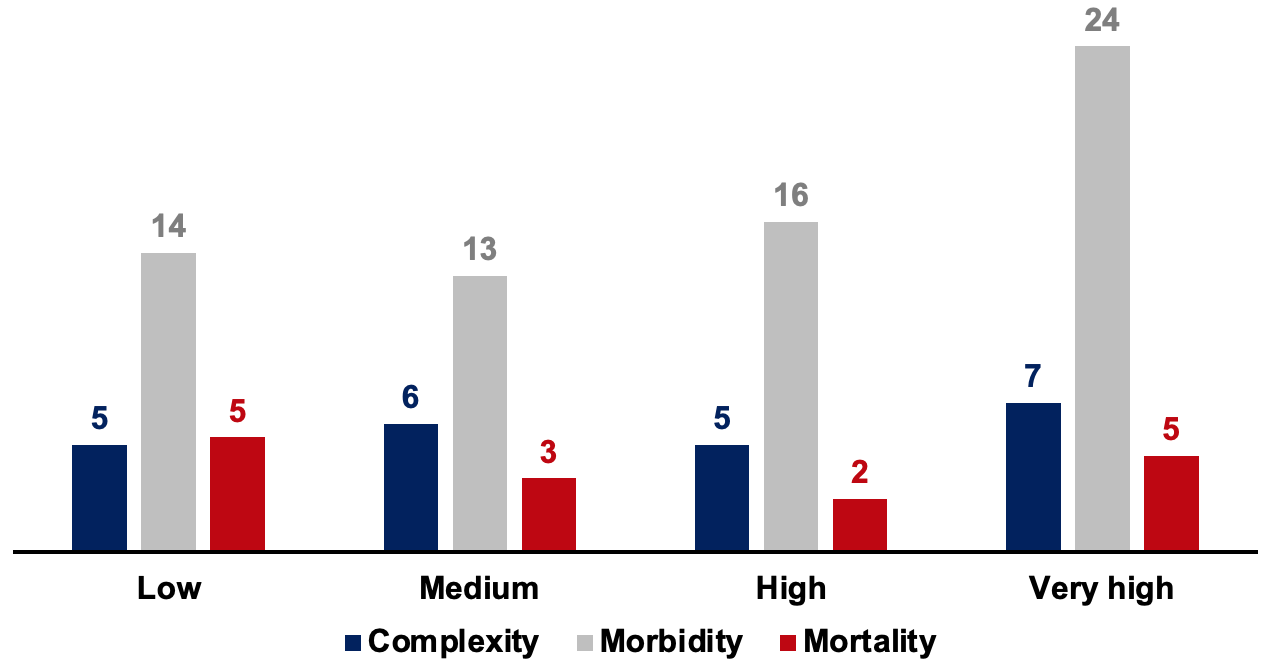


**Supplementary Figure 13.** Complexity of liver surgery, morbidity, and mortality among the groups of Total Health Expenditure


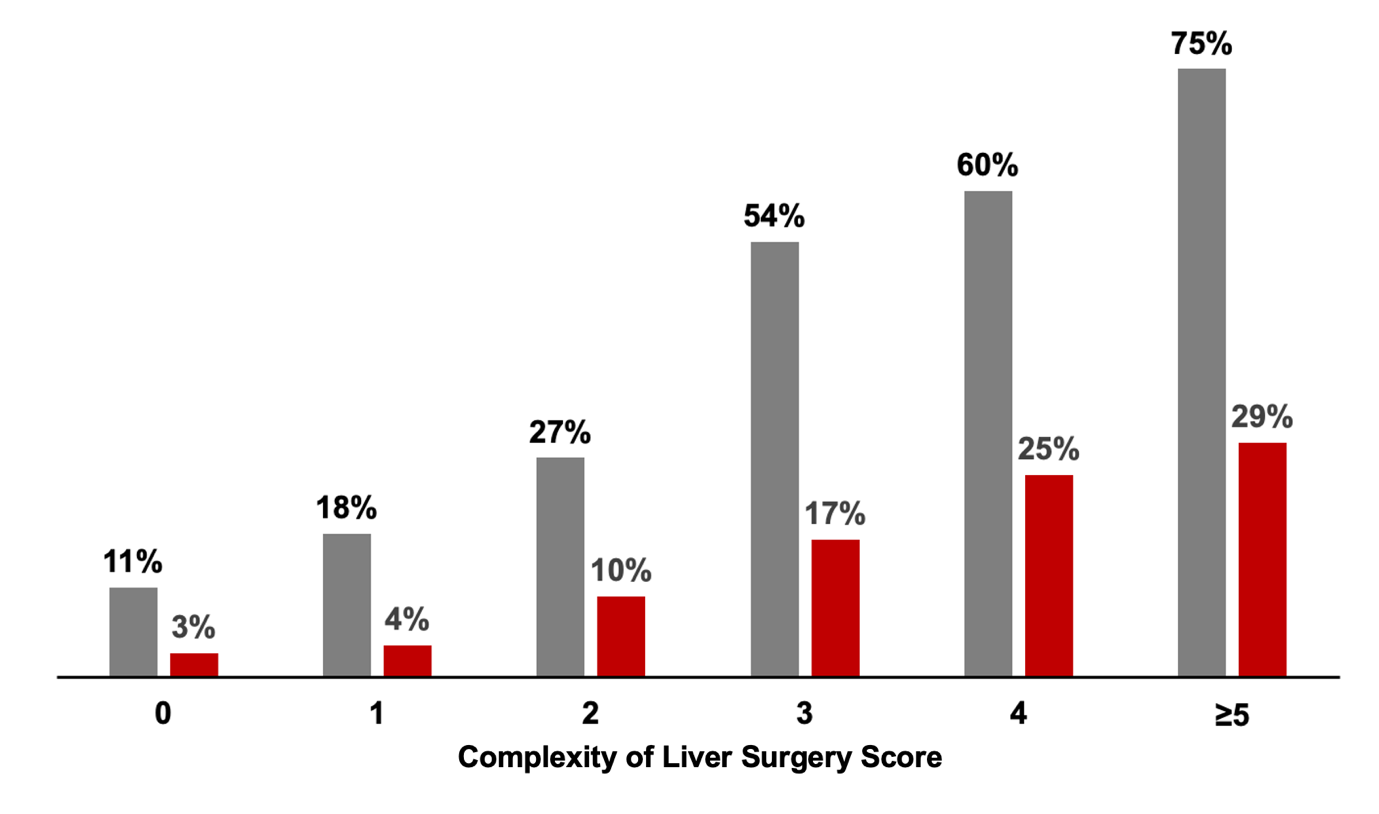


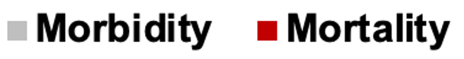


**Supplementary Figure 14.** Complexity of liver surgery associated with morbidity (**grey**) and mortality (**red**) rates.


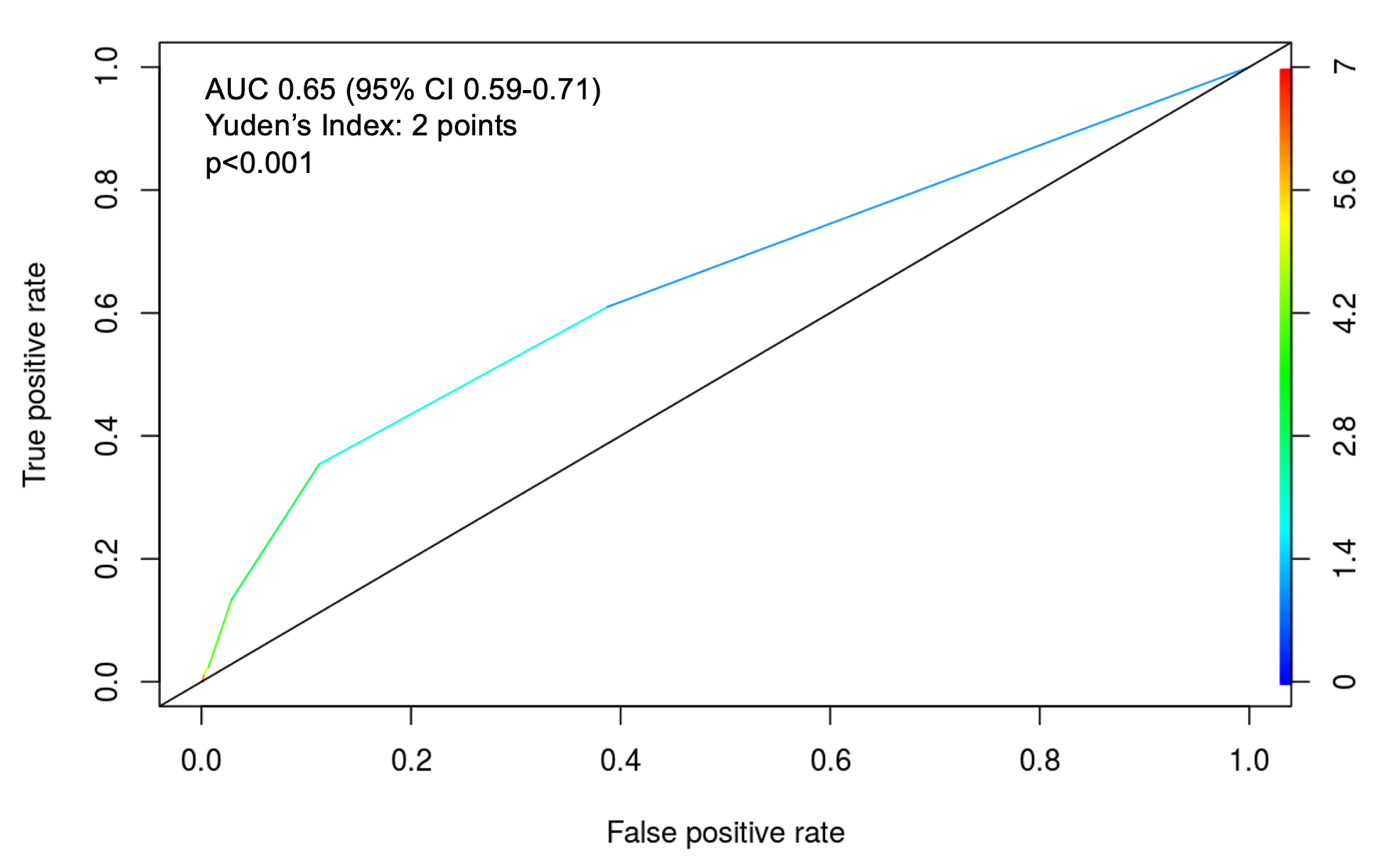


**Supplementary Figure 15.** Identification of the optimal cut-off point of the complexity of liver surgery score using ROC analysis and the Yuden’s index.


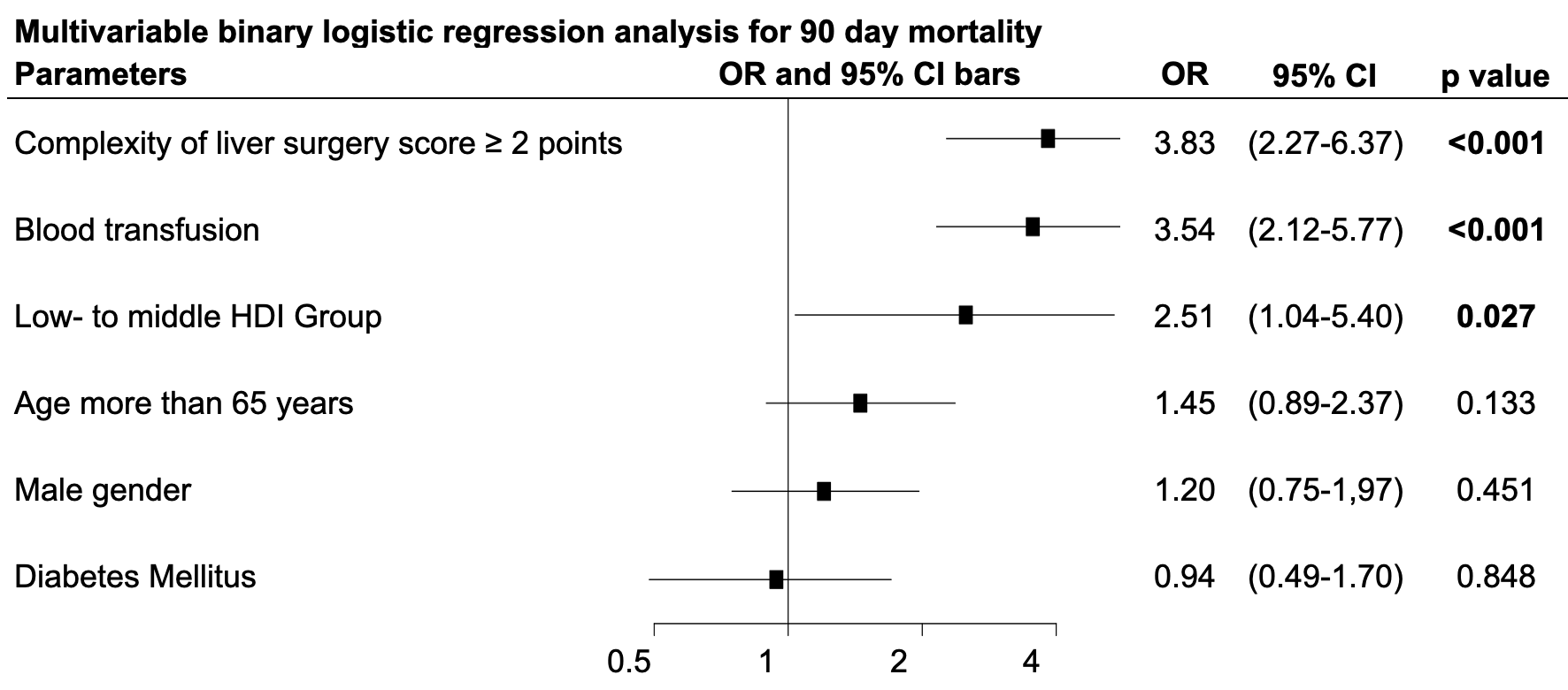


**Supplementary Figure 16.** Multivariable analysis for independent factors of 90-day mortality, including the complexity of liver surgery score.
