# Supplementary material for: Outcomes of elective liver surgery worldwide: a global, prospective, multicenter, cross-sectional study
Source: Int J Surg. 2023 Oct 4;109(12):3954–66. doi: 10.1097/JS9.0000000000000711 (PMC10720814; doi:10.1097/JS9.0000000000000711)
Supplement: SUPPLEMENTARY MATERIAL [file js9-109-3954-s002.docx]

**Supplemental tables**

**Supplemental table 1.** Patient characteristics, morbidity, and mortality rates for benign *versus* malignant disease

| **Parameters** | **Benign** | **Malignant** | **OR (95% CI)** | ***p* value** |
| --- | --- | --- | --- | --- |
|  | *n=374* | *n=1785* |  |  |
| Age, median (IQR) | 48 (34-62) | 66 (58-72) | - | **<0.001** |
| Female gender, n (%) | 232 (62) | 704 (39) | 2.51 (1.98-3.18) | **<0.001** |
| BMI, kg/m2, median (IQR) | 25 (22-28) | 26 (23-29) | - | **<0.001** |
| Coronary artery disease, n (%) | 19 (5) | 210 (12) | 2.50 (1.53-4.28) | **<0.001** |
| Diabetes mellitus, n (%) | 26 (7) | 342 (19) | 3.17 (2.08-5.01) | **<0.001** |
| COPD/Asthma, n (%) | 23 (6) | 129 (7) | 1.19 (0.75-1.97) | 0.506 |
| Liver surgery complexity score, mean (SD) | 0.3 (0.7) | 0.6 (0.9) | - | **<0.001** |
| CCI® at 90 days, median (IQR) | 0 (0-21) | 0 (0-21) | - | **0.003** |
| Complication of any severity, n (%) | 130 (35) | 782 (44) | 1.46 (1.15-1.86) | **0.001** |
| Major complication (Grade ≥3a), n (%) | 54 (14) | 287 (16) | 1.14 (0.82-1.59) | 0.483 |
| ICU stay, median (IQR) days | 1 (0-2) | 1 (0-2) | - | 0.483 |
| Hospital stay, median (IQR) days | 8 (5-11) | 8 (5-12) | - | 0.234 |
| Mortality at 90 days, n (%) | 10 (3) | 72 (4) | 1.52 (0.77-3.33) | 0.295 |
